# New sugar-derived compounds as inhibitors of carbon steel against corrosion in acid solutions: experimental analyses and theoretical approaches†

**DOI:** 10.1039/d5ra00835b

**Published:** 2025-05-19

**Authors:** M. Rbaa, A. Barrahi, R. Seghiri, Konstantin P. Katin, Elyor Berdimurodov, Hatem A. Abuelizz, C. Jama, F. Bentiss, B. Lakhrissi, A. Zarrouk

**Affiliations:** a The Higher Institute of Nursing Professions and Health Techniques of Casablanca P.O. Box 20250 Casablanca Morocco mohamed.rbaa10@gmail.com +212702099423; b Laboratory of Organic Chemistry, Catalysis and Environment, Faculty of Sciences, Ibn Tofail University PO Box 133 14000 Kenitra Morocco; c Laboratory of Materials, Nanotechnology, and Environment, Faculty of Sciences, Mohammed V University in Rabat P.O. Box 1014, Agdal Rabat Morocco azarrouk@gmail.com +212665201397; d Ecole Nationale Supérieure de Chimie de Kenitra (ENSCK), Ibn Tofail University Kenitra Morocco; e Laboratory of Advanced Materials and Process Engineering, Faculty of Sciences, Ibn Tofail University Kenitra Morocco; f National Research Nuclear University “MEPhI” KashirskoeShosse 31 Moscow 115409 Russian Federation; g Faculty of Chemistry, National University of Uzbekistan Tashkent 100034 Uzbekistan; h Department of Pharmacy and Chemistry, Alfraganus University Tashkent 100190 Uzbekistan; i Chemistry and Physics, Western Caspian University Baku AZ-1001 Azerbaijan; j Department of Pharmaceutical Chemistry, College of Pharmacy, King Saud University PO Box 2457 Riyadh 11451 Saudi Arabia; k Univ. Lille, CNRS, INRAE, Centrale Lille, UMR 8207, UMET – Unité Matériaux et Transformations F-59000 Lille France; l Laboratory of Catalysis and Corrosion of Materials, Faculty of Sciences, Chouaib Doukkali University PO Box 20 M-24000 El Jadida Morocco

## Abstract

The purpose of this study is to investigate the acid corrosion inhibition efficiency on carbon steel (CS) by utilizing two novel quinoxaline derivatives obtained from the reaction of recently synthesized d-mannose (MR_1_ and MR_2_) *via* nucleophilic substitution (SN_1_). The synthesized compounds were recently characterized by ^13^C-NMR and ^1^H-NMR spectroscopy. Electrochemistry testing was employed to evaluate their protective efficiency, whereas the surface was investigated using X-ray photoelectron spectroscopy (XPS) and scanning electron microscopy (SEM). The results indicate that the two inhibitors MR_1_ and MR_2_ exhibit inhibition efficiencies of 95.3% and 94.8% at 10^−3^ M for MR_1_ and MR_2_, respectively. The impedance results indicated that the incorporation of MR_1_ and MR_2_ into the corrosive medium reduces charge capacitance, hence systematically enhancing the interface charge/discharge function and creating an adsorbed layer on the metal surface. Moreover, SEM, water contact angle, and XPS techniques corroborated the formation of a protective coating on the carbon steel substrate surface following the incorporation of MR_1_ and MR_2_. The chemical interaction mechanisms at the atomic scale were analysed using theoretical calculations, DFT calculations and MD simulations.

## Abstract 2

The study used ^1^H-NMR and ^13^C-NMR to analyze structures. MR_1_ and MR_2_ showed inhibition efficiencies of 95.3% and 94.8% at 10^−3^ M. Surface analysis was done *via* XPS and SEM.

## Graphical abstract

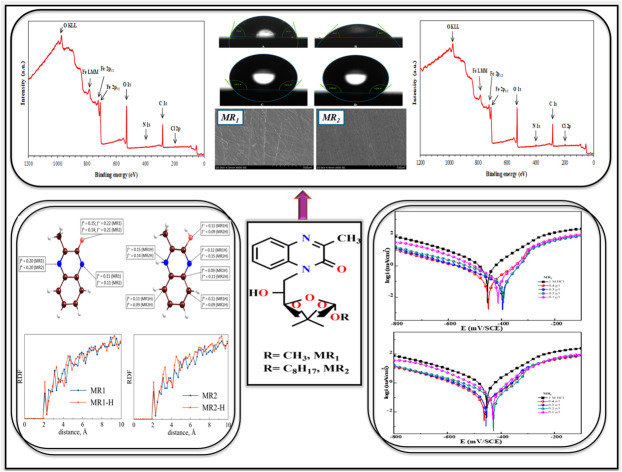

## 1. Introduction

Heterocycles are organic compounds characterized by a ring structure primarily composed of carbon atoms.^1^ Other elements, like oxygen (O),^2^ nitrogen (N),^3^ sulfur (S),^4^ phosphorus (P), and silicon (Si)^5^ may also be integrated into their structure as well. These compounds are widely found in nature, particularly in biological molecules such as amino acids,^6^ nucleotides,^7^ and vitamins.^8^ Furthermore, they are also used in scientific and industrial fields, including pharmacology,^9^ agrochemistry,^10^ materials science,^11^ and corrosion inhibition.^12–21^

Environmentally friendly organic compounds, also known as non-toxic compounds, are molecules derived from renewable natural raw materials or synthetically designed through specific processes, to reduce their environmental impact.^22,23^ These compounds are widely utilized across various industries,^24^ including cleaning agents, personal care products,^25^ food products,^26^ and building materials.^27^ The grafting of d-mannose onto organic compounds involves the formation of a covalent bond between a d-mannose molecule and an organic substrate, such as quinoline or quinoxaline.^28^ This chemical synthesis alters the structure of the original molecule or generates new compounds with biological or pharmaceutical properties.^28^

This study focuses on the synthesis of novel organic compounds derived from d-mannose and their characterization using various spectroscopic techniques, particularly, ^1^H-NMR and ^13^C-NMR nuclear magnetic resonance. The primary objective of synthesizing the MR_1_ and MR_2_ compounds is to explore their potential as corrosion inhibitors in an acidic environment (1.0 M HCl). To achieve this, a series of electrochemical analyses, including EIS as well as analytical investigations such as XPS, contact angle measurements and SEM, were conducted on both the corrosive solution and the metallic substrate surface of the steel.

## 2. Experimental details

### 2.1. Synthesis of compounds

All materials and reagents necessary for the synthesis of MR_1_ and MR_2_, used as corrosion inhibitors in this study, were procured from Sigma-Aldrich Chemical Company in France. The synthesized MR_1_ and MR_2_ compounds characterized through nuclear magnetic resonance spectroscopy (JNM-ECZ500R/S_1_-FT NMR System de JEO, DMSO-d_6_ reference at *δ*_ppm_). Reaction progress was observed by thin-layer chromatography on silica gel (TLC, 60 F254). Melting points (*M*_P_) was determined using a Kofler bench. The organic solvents employed for the reaction and subsequent product purification were purified by distillation.

### 2.2. Surface characterization

The morphology of CS was analyzed using scanning electron microscopy (SEM) on a JEOL 5300 microscope with a 5 kV, enabling the examination of the substrate in the absence and presence of MR_1_ and MR_2_ inhibitors.

Surface wettability was assessed by measuring water contact angles using a DSA100 drop shape analyzer (Krüss, Germany). Samples measuring of 2 × 2 × 0.3 cm^3^ were tested with 2 μL drops. The surface free energy (SFE) was determined using three polar solvents: pure deionized water, formamide, and diiodomethane (purity > 99%, Sigma-Aldrich).^13,29^

X-ray photoelectron spectroscopy (XPS) was conducted on a KRATOS AXIS Ultra DLD spectrometer equipped with a monochromated Al Kα X-ray source (*hv* = 1486.6 eV) and a 1 mm beam diameter. Measurements were performed at a constant pass energy of 40 eV over an analysis area of 700 μm × 300 μm under an ultra-high vacuum 10^−10^ torr. Charge compensation was applied to mitigate electrostatic effects. Spectral deconvolved was carried out using a non-linear least-squares fitting approach with a Shirley baseline and a Gaussian–Lorentzian peak shape function. Data processing was performed using CasaXPS software.

### 2.3. Electrochemical measurements

The steel used in this study had a chemical composition that is composed of 0.370% (C), 0.059% (Ni), 0.230% (Si), 0.016% (S), 0.077% (Cr), 0.011% (Ti), and 0.680% (Mn), with the remaining mass percentage consisting of iron (Fe). Prior to each experiment, the steel samples were abraded using silicon carbide (SiC) abrasive paper with grit sizes ranging from 180 to 1200. The samples were then polished, rinsed with distilled water, and allowed to air dry at room temperature. Analytical-grade hydrochloric acid (HCl, 37% purity) was diluted with distilled water to prepare corrosive solutions with a concentration of 1 M HCl. The evaluated concentrations of the MR_1_ and MR_2_ inhibitors varied from 0.1 to 0.4 g L^−1^.

The electrochemical measurements were performed using a PGZ/100 potentiostat and Volta Master 4.0 software and three-electrode cell consisting of a carbon steel working electrode, a saturated calomel reference electrode (SCE) and a platinum counter electrode. The carbon steel reached a steady open circuit potential after 1800 seconds of immersion in the test solutions. Electrochemical impedance spectroscopy (EIS) was then performed at the open circuit potential (*E*_OCP_) with an AC signal amplitude of 10 mV frequency range of 100 kHz to 10 mHz. The data obtained were analyzed using the Zview software. Carbon steel, both with and without inhibitors, was then tested by potentiodynamic polarization experiments in 1 M HCl solutions. Potentials ranging from −800 mV/SCE to −100 mV/SCE were scanned at a rate of 0.5 mV s^−1^. The linear segments of the anodic and cathodic Tafel curves were extrapolated to obtain the current densities and associated electrochemical parameters. Data processing for polarization measurements was carried out using EC-Lab software.

### 2.4. Computational details

Two inhibitor molecules, MR_1_ and MR_2_, were analyzed computationally using DFT and MD methods. Both the neutral (MR_1_ and MR_2_) and protonated (MR_1_-H, MR_2_-H) forms of the inhibitors were considered. The protonated forms include two additional hydrogen atoms, attached to the nitrogen and to =O group. DFT calculations were performed using the B3LYP hybrid functional and 6-311G* basis set. Geometry optimization was conducted without symmetry constrains using the GAMESS-US program.^30^ The influence of solvent (water) was incorporated through the polarized continuum model in GAMESS-US.^31^

The behavior of MR_1_ and MR_2_ on the steel surface in a corrosive media was simulated using a realistic ReaxFF interatomic potential suitable for corrosion modeling.^32^ The surface Fe (110) was represented by 6-layers cell (approximately 1.2 nm thickness) consisting of 1440 Fe atoms (3 nm × 5 nm). The corrosive media was explicitly represented by 1000 H_2_O and 18 dissociated HCl molecules.^32^ Various initial orientations of the inhibitor molecule were tested to determine the most stable adsorption position. Molecular dynamics simulations were done employing the NVT ensemble at room temperature (300 K) with the LAMMPS program.^33^ The total simulation time was 1 ns, with a time step of 0.2 fs for Verlet integration of the equations motion.

## 3. Results and discussions

### 3.1. Organic synthesis

The organic compounds used in this study were synthesized through a three-step process. The initial step involved the synthesis of epoxy d-mannose, which has been previously detailed in a separate publication.^13^ The next step focused on the preparation of quinoxaline derivatives, a method that has also been documented in another manuscript.^34^

The final step involved grafting epoxy d-mannose with quinoxaline derivatives. This reaction was conducted by adding 0.85 eq. of quinoxalinone to a solution of the activated substrate in anhydrous DMF (33 g L^−1^) at 110 °C, in the presence of 1.2 eq. of K_2_CO_3_ and 0.1 eq. of BTBA. The resulting mixture was filtered, and then, the solvent was evaporated. Finally, the product was moved to silica gel chromatography for purification.

**Fig. 1:**
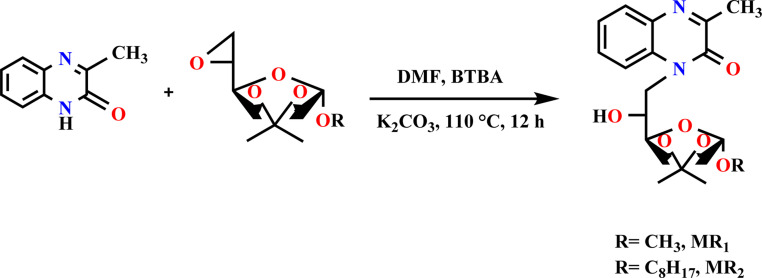
Preparation method for MR_1_ and MR_2_ compounds.

The progress of the reaction was monitored by TLC (eluent: hexane–acetone, 9 : 1, v/v, 12 hours). The compounds were purified through silica gel chromatography (eluent: hexane–acetone, 9.5 : 0.5, v/v), and their purity was purified by chromatographic. Their structures were confirmed by ^13^C-NMR, ^1^H-NMR (Fig. 1).

**Fig. 2:**
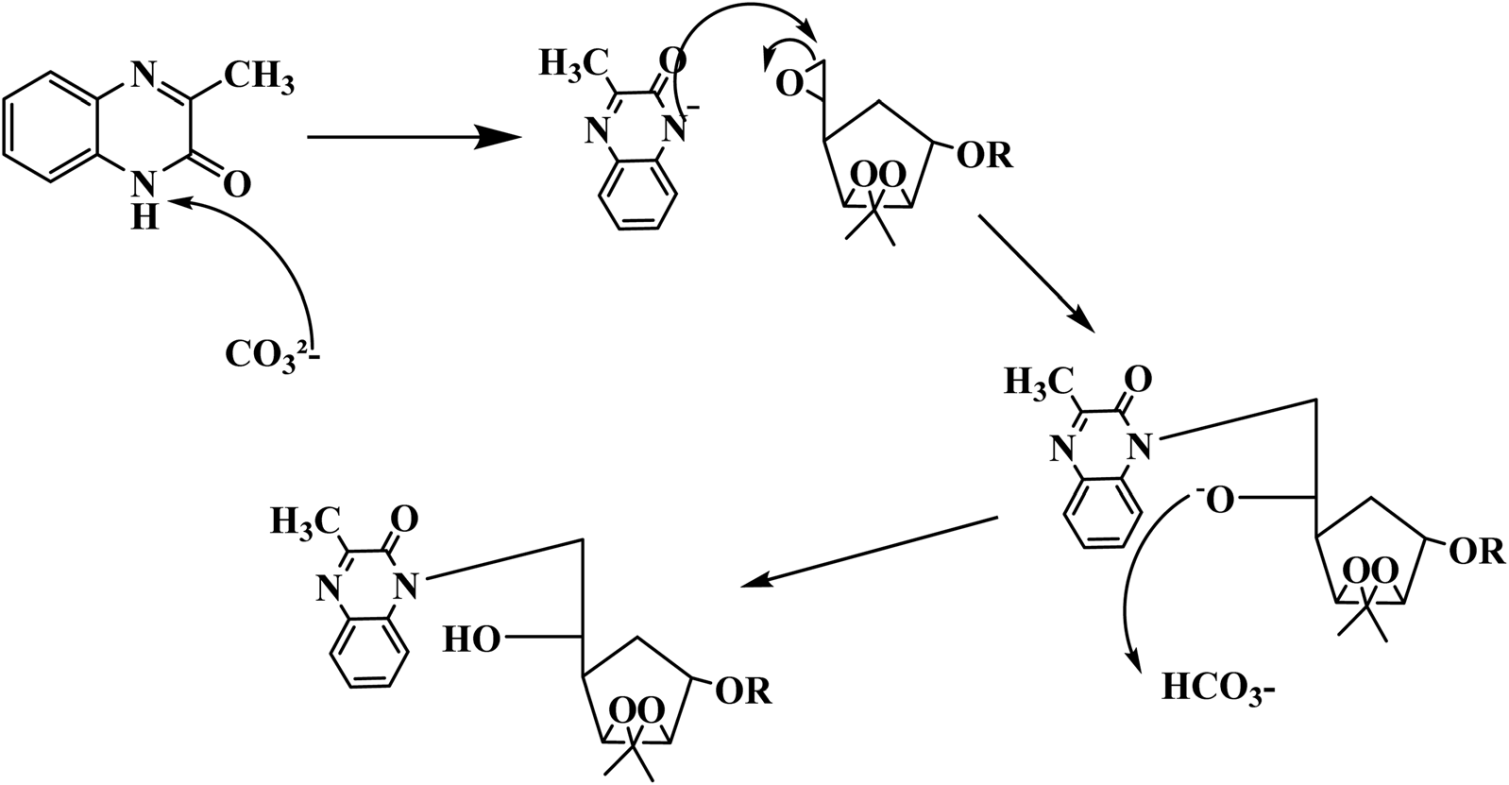
Proposed synthetic mechanism of the two compounds MR_1_ and MR_2_.

The mechanism of the two compounds is illustrated in Fig. 2.

### 3.2. Spectroscopic analysis of MR_1_ and MR_2_

#### 3.2.1 1-*N*-(6-Deoxy-2,3-*O*-isopropylidene-α-d-mannofuranoside of methyl-6-yl)-3-methylquinoxalinones (MR_1_)

Chemical formula: C_19_H_24_N_2_O_6_, *M* = 376.16 g mol^−1^, *Y* (%): 74, *M*_p_ = 136.00, aspect: white solid. ^1^H-NMR (*δ*_ppm_): 1.222 (s, 6H, 2CH_3-glucose_), 2.458 (d, 3H, CH_3-quinoxaline_), 3.706 (s, 3H, O–CH_3-glucose_), 3.742–5.819 (m, 8H, CH_-glucose_), 6.919–10.756 (m, 4H, ArH_-quinoxaline_), ^13^C NMR (*δ*_ppm_):26.305 (C–2C̲H_3-glucose_), 28.248 (O–C̲H_3-glucose_), 27.082 (C–CH_3-quinoxaline_), 57.853–111.267 (C̲H–C̲H_2-glucose_), 120.987–130.732 (ArC̲–ArC̲H_quinoxaline_), 154.709 (–N=C̲ar), 154.235 (–C̲=O).

#### 3.2.2 1-*N*-(6-Deoxy-2,3-*O*-isopropylidene-α-d-mannofuranoside of octyl-6-yl)-3-methylquinoxalinones (MR_2_)

Chemical formula: C_27_H_41_N_2_O_6_, *M* = 489.30 g mol^−1^, *Y* (%): 78, *M*_p_ = 120.00, aspect: liquide sirupeux. ^1^H-NMR (*δ*_ppm_): 1.195 (s, 6H, 2CH_3-glucose_), 2.044 (d, 3H, CH_3-quinoxaline_), 3.299 (s, 3H, O–CH_3-glucose_), 3.709–4.858 (m, 8H, CH_-glucose_), 5.723–8.214 (m, 4H, ArH_-quinoxaline_), ^13^C NMR (*δ*_ppm_):19.301 (C–2C̲H_3-glucose_), 19.327 (O–C̲H_3-glucose_), 39.305 (C–CH_3-quinoxaline_), 61.315–82.007 (C̲H–C̲H_2-glucose_), 97.439–111.265 (ArC̲–ArC̲H_quinoxaline_), 163.580 (–C̲=O).

### 3.3. OCP analysis

**Fig. 3:**
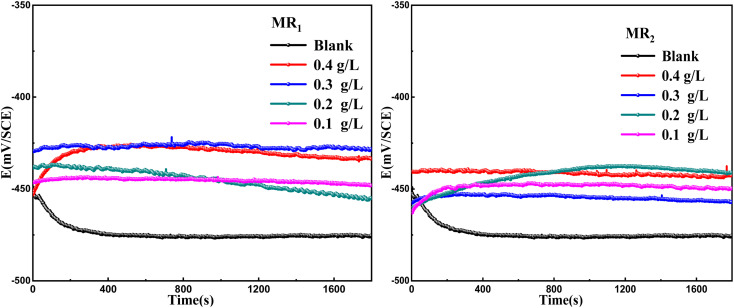
The time-dependent variation of OCP for carbon steel in 1 M HCl at 303 K with and without the different concentrations of MR_1_ and MR_2_.

Fig. 3 illustrates the variation in open circuit potential (*E*_OCP_) of carbon steel in a 1.0 M HCl solution with and without different concentrations of MR_1_ and MR_2_ inhibitors at 303 K. The curves exhibit a shift toward more positive potentials, indicating that the addition of MR_1_ and MR_2_ inhibitors significantly influences the electrochemical behavior of carbon steel. The open circuit potential shifted anodically, suggesting a slowdown in the anodic dissolution of iron. This finding implies that MR_1_ and MR_2_ form a protective layer on the metal surface, effectively mitigating corrosion in hydrochloric acid. Furthermore, after approximately 30 minutes of immersion, the potential stabilized, indicating that oxidation and reduction processes had reached equilibrium at the electrode–solution interface. This immersion time was used as a reference for subsequent electrochemical experiments.

### 3.4. EIS analysis

The EIS is a technique employed for observing the deterioration of steel, particularly carbon steel. In this study, we applied this approach while examining the impact of organic inhibitors obtained from 8-hydroxyquinoline grafted by d-mannose MR_1_ and MR_2_ at 0.1, 0.2, 0.3 and 0.4 g L^−1^, both with and without their presence.

**Fig. 4:**
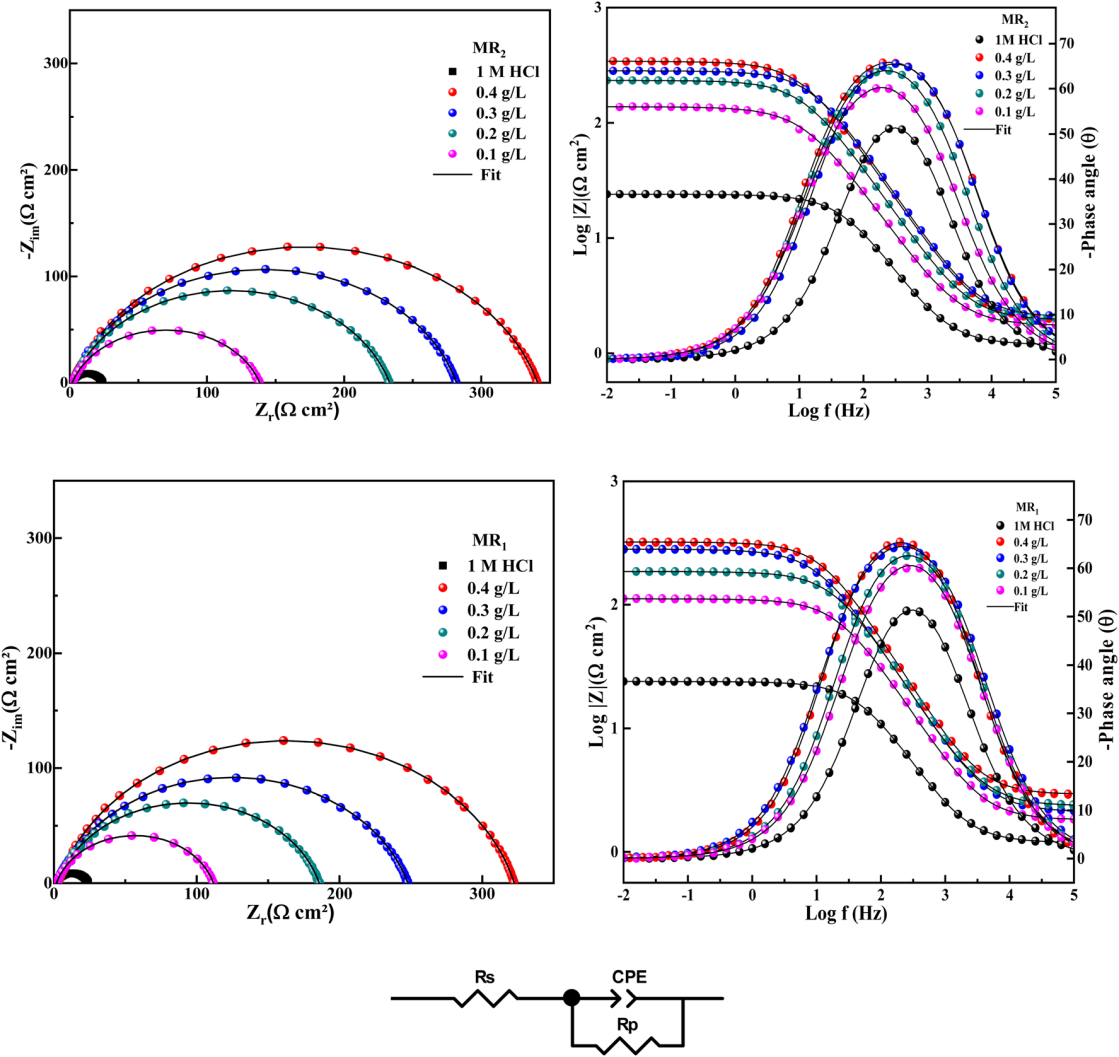
The Nyquist, Bode and phase plots were constructed for CS after in 1 M HCl, with and without different concentrations of MR_1_ and MR_2_ and the equivalent circuit used.

Fig. 4 illustrates the Nyquist impedance electrochemical spectra for the two organic inhibitors obtained from 8-hydroxyquinoline grafted by d-mannose MR_1_ and MR_2_ in comparison with the control (1 M HCl). The recorded spectra exhibit slightly diminished semicircles, suggesting that the corrosion of steel in 1 M HCl occurs *via* a charge transfer mechanism.^35^ The semicircles diameter expands following the introduction of the two organic inhibitors, derived from 8-hydroxyquinoline grafted by d-mannose MR_1_ and MR_2_, compared to the solution only hydrochloric acid (HCl). At lower frequencies, the capacitive loop surpasses the Nyquist semicircles, indicating surface heterogeneity on the carbon steel (CS) due to the adsorption of MR_1_ and MR_2_ onto the metallic surface (CS).

Moreover, the obtained curves exhibit similarity upon the addition of MR_1_ and MR_2_, suggesting that the corrosion mechanism remains unchanged.^35^

In Fig. 4, the examination of the Bode and phase diagrams reveals a shift in the phase angle values. The phase angle value peaks at a moderate frequency, signifying the presence of a single charge transfer process.^36^

A singular time constant and a streamlined comparable circuit were utilized to examine the EIS data. The electrochemical values obtained from this approach are detailed in Table 1. A constant phase element (CPE) representing the double layer capacitance (*C*_dl_) on a heterogeneous surface is one of these attributes, alongside *R*_p_ (polarization resistance) and *R*_s_ (solution resistance). Additionally, the equations presented below (1) (ref. 37 and 38) were employed to quantify the impedance of the constant-phase element (*Z*_CPE_).1
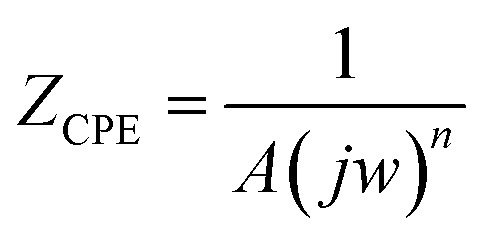
In this context, *j* denotes the imaginary unit, *A* signifies the CPE constant, and *n* represents the angular frequency. The *C*_dl_ values were ascertained using eqn (2):^39,40^

2 *C*_dl_=(*A* × *R*_P_^1−*n*^)^1/*n*^

**Table 1:** EIS parameters for MR_1_ and MR_2_

Inhibitor	Conc. (g L^−1^)	*R*_s_ (Ω cm^2^)	*R*_p_ (Ω cm^2^)	10^6^ × *A* (Ω^−1^ s^*n*−1^ cm^−2^)	*n*_dl_	*C*_dl_ (μF cm^−2)^	*χ* ^2^	*η*_EIS_%
1 M HCl	—	0.83	21.57	293.9	0.845	116.2	0.002	—
MR_2_	0.1	1.8	126.2	170.9	0.83	77.8	0.006	82.9
	0.2	2.0	231.1	113.2	0.83	53.7	0.005	90.7
	0.3	2.1	279.6	96.8	0.84	48.7	0.006	92.3
	0.4	1.9	343.5	71.4	0.85	37.1	0.009	**93.7**
MR_1_	0.1	1.9	109.9	149.8	0.82	60.8	0.004	80.3
	0.2	2.3	183.4	121.8	0.83	55.9	0.009	88.2
	0.3	2.4	244.5	97.2	0.84	47.7	0.008	91.2
	0.4	3.0	319.5	80.7	0.84	40.2	0.007	**93.2**

The inhibition efficiency (*η*_EIS_ (%)) was determined using the subsequent formula(3):^41^3
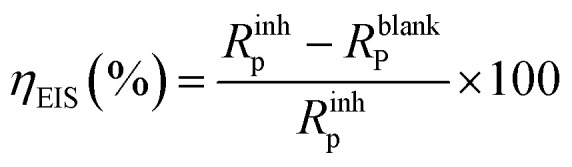
where *R*^inh^_p_ and *R*^blank^_P_ represent the polarization resistance after to and prior to the introduction of the inhibitor, respectively. Since we worked under the same conditions for both stationary and transient polarisation techniques, we referred to our team's results concerning the effect of concentration without the inhibitor.^42^

The fitted values are displayed in Table 1, according to the circuit equivalent illustrated in Fig. 4. This circuit comprises solution resistance (*R*_s_), polarization resistance (*R*_p_), and the constant and exponent of the constant phase element (CPE), denoted as *A* and *n*.

The findings in Table 1 demonstrate that when the concentration of the inhibitors MR_1_ and MR_2_, which are derived from 8-hydroxyquinoline grafted onto d-mannose, increases, the polarization resistance (*R*_p_) gradually increases. This increase in *R*_p_ is accompanied by a significant decrease in double layer capacitance (*C*_dl_), which reflects the replacement of the water molecules initially adsorbed by the MR_1_ and MR_2_ inhibitor molecules on the metal surface.^43^ This substitution reduces the dielectric constant and promotes the formation of an electrical double layer, enhancing inhibitor adsorption. MR_2_ outperforms MR_1_ with a maximal inhibitory efficiency (*η*_EIS_) of 93.7% *vs.* 93.2%.

The *R*_p_ values increase as a result of the inhibitor molecules adhering to the surface exposed to the corrosive environment and blocking active corrosion sites. This interaction also leads to an increase in the deflection parameter (*n*), which is associated with a reduction in surface defects, as well as a significant decrease in the admittance (*Q*) of protected substrates compared to untreated ones. Increasing surface coverage (*θ* = *η* (%)/100) leads to a linear decrease in *C*_dl_, indicating effective molecular adsorption and limiting metal dissolution. Furthermore, the strong bonds between the active components (MR_1_ and MR_2_) and the metal are made possible by the presence of free electron pairs on the heteroatoms and the d-orbitals, which enhance inhibitor–molecule interactions with the metal surface. This process slows down the rate of corrosion of the metal by blocking the active corrosion sites. Finally, the reliability of the equivalent circuit used to describe the electrochemical interactions is confirmed by the chi-square (*χ*^2^) values, which are around 10^−3^.

### 3.5. Potentiodynamic polarization studies

**Fig. 5:**
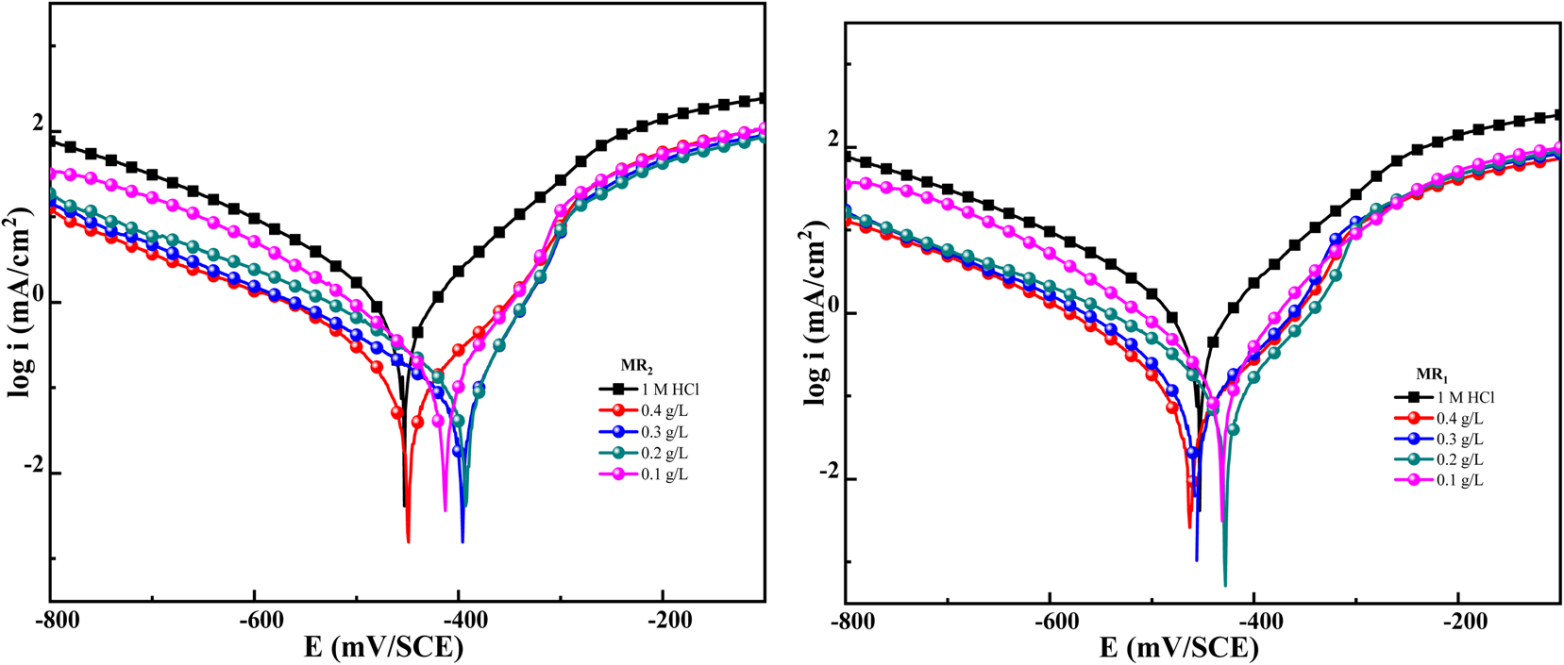
Tafel plots of MR_1_ and MR_2_.

In these studies, key Tafel parameters were found. Additionally, this method allows for the classification of the inhibitor as either anodic, cathodic, or a combination of both, as illustrated in Table 2 and Fig. 5. Inhibitory efficacy was calculated using the following relationship:4
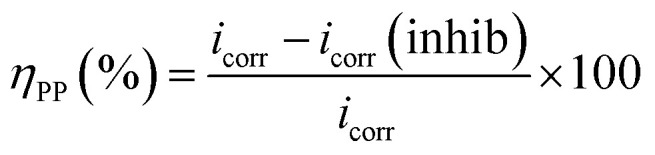
*i*_corr_ and *i*_corr(inhib)_ were characterized as corrosion current densities in the absence and presence of MR_1_ and MR_2_.

**Table 2:** Tafel results of MR_1_ and MR_2_

Inhibitor	Conc. (g L^−1^)	*E*_corr_ (mV *vs.* SCE)	*i*_corr_ (μA cm^−2^)	−*β*_c_ (mV dec^−1^)	*β*_a_ (mV dec^−1^)	*η*_pp_ (%)
1 M HCl	—	456.3	1104	155.4	112.8	—
MR_2_ 1800	0.1	−415.2	161.1	135	98	85.4
	0.2	−394.7	97.6	128	85	91.2
	0.3	−400.1	72.4	121	84	93.4
	0.4	−447.8	51.4	118	68	**95.3**
MR_1_	0.1	−429.2	191.0	138	83	82.7
	0.2	−428.4	128.5	129	76	88.4
	0.3	−457.0	75.8	123	78	93.1
	0.4	−461.1	57.2	105	75	**94.8**

Fig. 5 shows a shift in the Tafel curves towards lower potentials when the d-mannose derivatives derived from quinoxaline MR_1_ and MR_2_ were introduced into the corrosive media. This shift indicates that the inhibitors were effective in reducing the electrochemical processes involved in corrosion. Specifically, MR_1_ and MR_2_ inhibited both the anodic dissolution of iron and the cathodic hydrogen evolution reaction when added to a 1 M HCl solution.

Table 2 shows that the inclusion of d-mannose derivatives derived from quinoxaline MR_1_ and MR_2_ resulted in a significant drop in corrosion current density (*i*_corr_), which is consistent with the polarization curves. The inhibition efficiency (*η*_pp_) reached 95.3% for MR_2_ and 94.8% for MR_1_ at 0.4 g L^−1^, indicating successful inhibition of electrochemical processes and the formation of a durable protective coating on the steel surface.^44^

Furthermore, the variation in corrosion potential (*E*_corr_) after the addition of the inhibitors remained minimal, at less than 85 mV. This suggests that MR_1_ and MR_2_ act as mixed inhibitors, affecting both anodic and cathodic processes.^45^ The cathodic polarization curves show parallel lines and slight variations in the cathodic Tafel slope (*β*_c_), indicating that the hydrogen evolution reaction is controlled but less intense due to inhibitor coverage on the steel surface. This inhibition is generated by the adsorption of MR_1_ and MR_2_ molecules on the substrate surface of carbon steel, which prevents the active sites from engaging in corrosion reactions. As a result, the decreases H^+^ ion access to the metal surface is restricted without affecting the cathodic reaction process. The surface coverage reduces the number of reactive sites available for H^+^ ions, slowing the cathodic reaction. In addition, the lower current densities observed in the anodic region indicate a slower rate of decrease in corrosion rate. The inhibitors MR_1_ and MR_2_ form a protective coating that reduces the severity of anodic iron dissolution, thereby enhancing the steel's resistance to corrosion.

### 3.6. Adsorption isotherm

To better understand the adsorption behaviour of d-mannose derived compounds MR_1_ and MR_2_ on the studied steel surface, some models were explored, including Langmuir, Frumkin, Freundlich, and Temkin. These isotherms serve to characterize the interaction behavior between the inhibitors and the surface, distinguishing between electrostatic adsorption (physisorption) or chemical adsorption (chemisorption).^46^

**Fig. 6:**
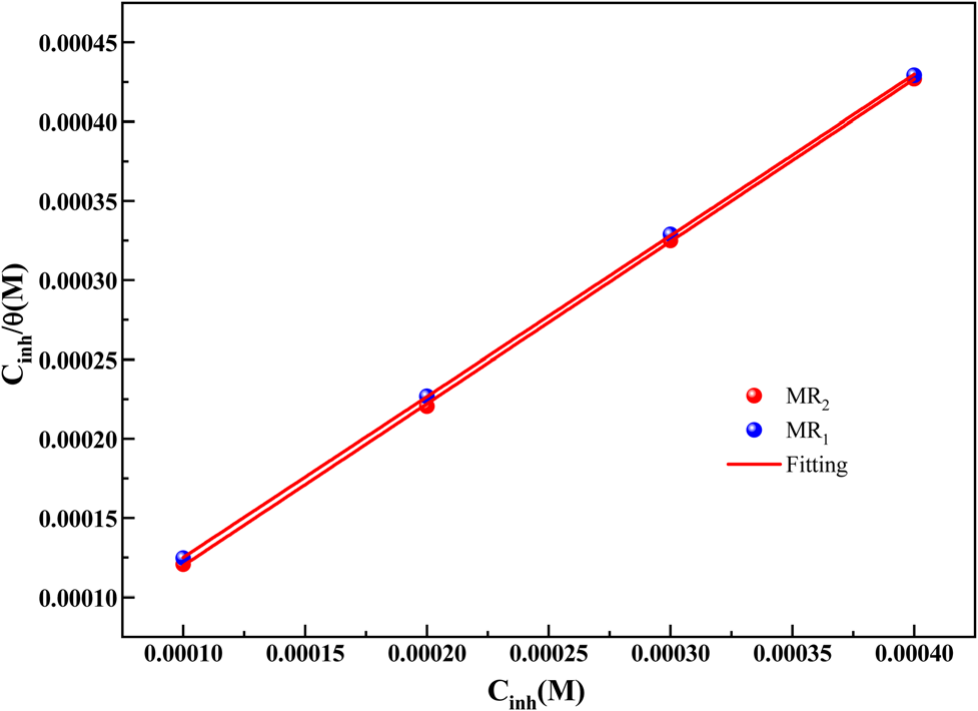
Langmuir model of MR_1_ and MR_2_.

The association between the derivatives of d-mannose MR_1_ and MR_2_ and the metal surface is presented in Fig. 6. The visual representation in the figure clearly validates that the Langmuir model is the most suitable for these inhibitors. The values of the adsorption constant (*K*), free enthalpy (Δ*G*_ads_), and correlation factors (*R*^2^) derived from the Langmuir isotherm for the d-mannose derivatives MR_1_ and MR_2_ are presented in Table 3.

**Table 3:** The obtained values of the adsorption isotherms

Inhibitor	*K* × 10^4^ (L mol^−1^)	Δ*G*_ads_ (kJ mol^−1^)	Slope	*R* ^2^
MR_2_	5.74	−37.7	1.02332	0.9999
MR_1_	4.29	−37.0	1.01614	0.9999

The Langmuir isotherm was used to calculate the inhibitors adsorption coefficient *K*, which was found to be 5.74 × 10^4^ L mol^−1^ for MR_2_ and 4.29 × 10^4^ L mol^−1^ for MR_1_. These high values indicate a strong affinity between the inhibitors. Effective adsorption is shown by a high adsorption coefficient, signifying that the inhibitor molecules cover a large surface area and keep corrosive species like Cl^−^ ions from getting to the steel surface.

The good adsorption of the inhibitors can be attributed to the displacement of adsorbed water molecules by the MR_1_ and MR_2_ leading to the formation of a stable protective layer. This layer serves as a barrier to the diffusion of corrosive species, significantly decreasing the corrosion rate. Furthermore, the presence of free electron pairs on the heteroatoms (nitrogen and oxygen) promotes chemical interactions, along with interactions with the metal's d-orbitals, resulting in strong bonds between the inhibitors and the surface. The difference in *K*_ads_ values between MR_2_ and MR_1_ indicates that MR_2_ exhibits a stronger affinity for the metal surface, resulting in superior inhibitory efficiency (95.3% *vs.* 94.8%).

The calculated Δ*G*_ads_ values are negative, ranging from −37.7 kJ mol^−1^ to −37.0 kJ mol^−1^. According to the literature, a free enthalpy value equal to or exceeding −40 kJ mol^−1^ an adsorption mechanism involving a both physisorption and chemisorption nature of the MR_1_ and MR_2_.^47,48^

The outcomes confirm the strong adhesion of the glucose-derived inhibitors MR_1_ and MR_2_ to the steel surface, providing long-term corrosion prevention, even in demanding industrial conditions.^29^

The findings we obtained are in agreement with literature values with respect to both inhibition efficiencies and adsorption behaviors. For example, Lei Guo *et al.* have looked at a newly synthesized triazolopyrimidine derivative as a corrosion inhibitor for toward mild steel in molar HCl. In their study, the reported inhibition efficiency was around 96.2% for the optimum concentration measured by impedance spectroscopy, and 93.7% at the optimal concentration using potentiodynamic polarization measurements.

In comparison, we obtained inhibition efficiencies *via* impedance spectroscopy of 93.7% for the MR_2_ and 93.2% for the MR_1_, and using potentiodynamic polarization measurements, we obtained 95.3% and 94.8% inhibition efficiencies for MR_2_ and MR_1_, respectively.

Both our compounds and those of by Lei Guo *et al.* adsorb to the steel surface *via* the Langmuir isotherm, which would suggest a similar adsorption mechanism.^49^

### 3.7. Surface analysis

To validate the adsorption of d-mannose derivatives on metal surfaces, a series of surface characterization analyses were carried out. These analyses involved the use of SEM, contact angle measurements and XPS.^50^

#### 3.7.1. SEM analysis

**Fig. 7:**
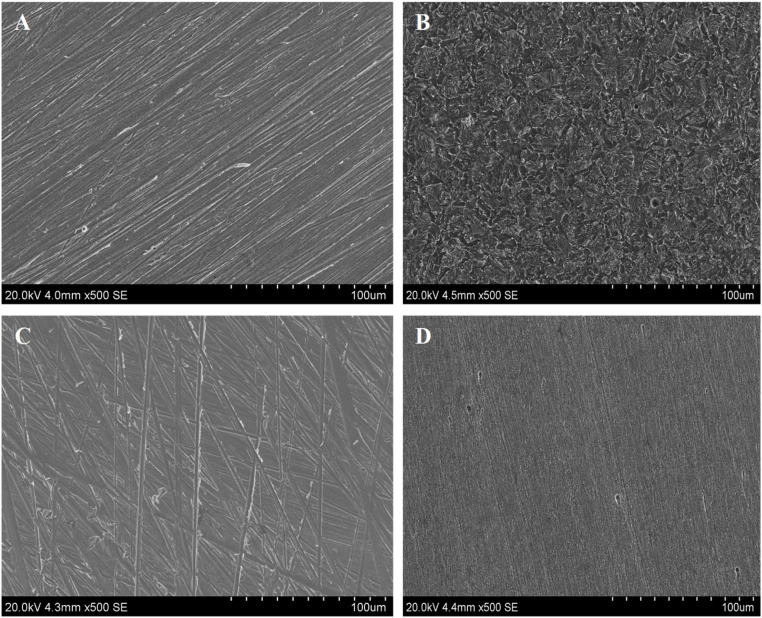
SEM images of M-steel surface were captured at different stages: (A) untreated metal surface, (B) immersed surface, with (C) MR_1_, and (D) MR_2_ at 400 ppm.

A comparative analysis of the steel surface images, both without (Fig. 7A and B) and coated with d-mannose derivatives MR_1_ (Fig. 7C) and MR_2_ (Fig. 7D) clearly illustrates a significant improvement in the metal's surface. The observations reveal oxidation phenomena, including pitting and cracking, along with the accumulation of iron oxides and hydroxides on active sites. The high solubility of oxygen in water promotes iron oxidation. In comparison with protected system, the metal surface appears smoother, suggesting that oxidation was inhibited by the formation of a Me-inhibitor complex at active sites.^51^

#### 3.7.2. Contact angle measurements

**Fig. 8:**
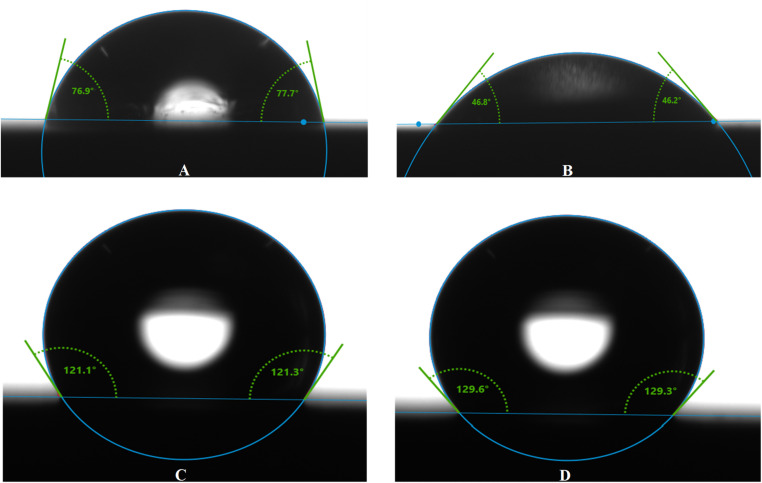
Images of the surface contact angle for selected metal: (A) before, (B) after corrosion, (C) with MR_1_, and (D) with MR_2_.

To identify the hydrophobic nature of metals surface after the protection and corrosion processes. The estimated values of contact angle reveal the hydrophobicity of selected the studied system. The corrosion inhibitor plays a critical role in controlling the surface's hydrophilicity, which directly impacts the effectiveness. He corrosion protection also depends on the hydrophobicity. The contact angle analysis enabled the assessment of the steel surface wettability and the efficiency of the adsorbed inhibitor layers. It was indicated that the contact angle was around 77° for metal sample, showing the moderate hydrophilicity (Fig. 8).

When the metal surface interacted with the HCl solution, this value was decreased to around 46°, which can be attributed to the formation of more hydrophobic compounds on the surface due to the metal–oxidation processes, such as the formation of Fe_2_O_3_ and Fe_3_O_4_. These oxidation products can reduce hydrophilic nature. These products develop the surface's efficiency for the corrosive medium, promoting the spread of droplets and accelerating the corrosion process.

The introduction of MR_1_ and MR_2_, resulted in changes to the contact angle, which increased to 121.2° for MR_1_ and 129.5° for MR_2_ (Fig. 8). This increase indicates a higher degree of surface hydrophobization, suggesting the formation of an effective organic barrier that protects from the water droplets and corrosion attacks.

The surface hydrophobicity increases with the adsorption of MR_1_ and MR_2_, which linked with the surface through the –OH, –C=O, –N= and aromatic groups. These functional groups are more hydrophilic and formed the rigid covalent bonds between surface and MR_1_ and MR_2_. In contrast, the hydrophobic alkyl chains of MR_1_ and MR_2_, especially the longer chain in MR_2_, enhance the surface hydrophobicity. The MR_2_ based protective film is more hydrophobic than that of MR_1_ due to the long alkaline chain of MR_2_, which supports the hydrophobicity of surface, providing better surface coverage and more effective blocking of corrosive ions.^52–54^

#### 3.7.3. XPS analysis

**Fig. 9:**
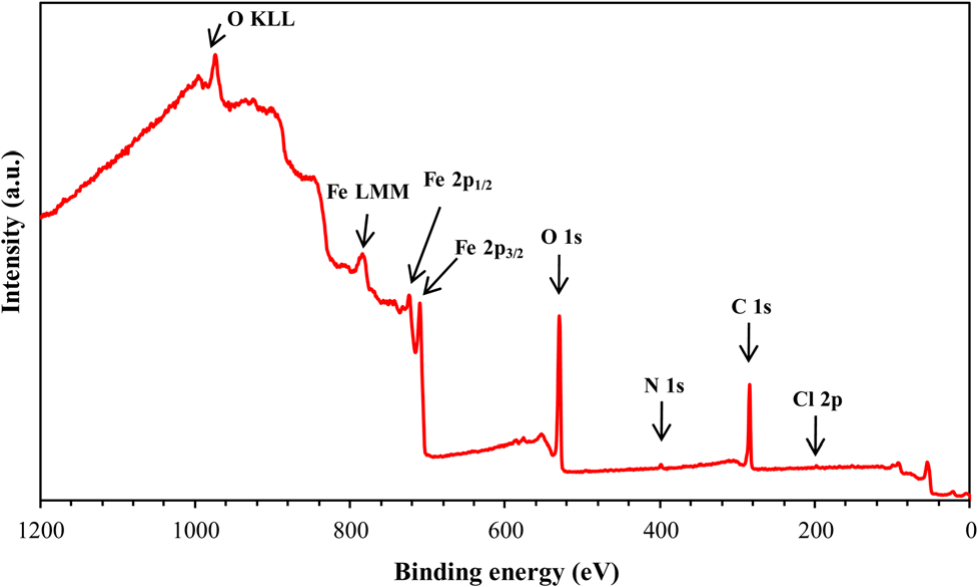
XPS spectrum of MR_1_/treated CS surface.

**Fig. 10:**
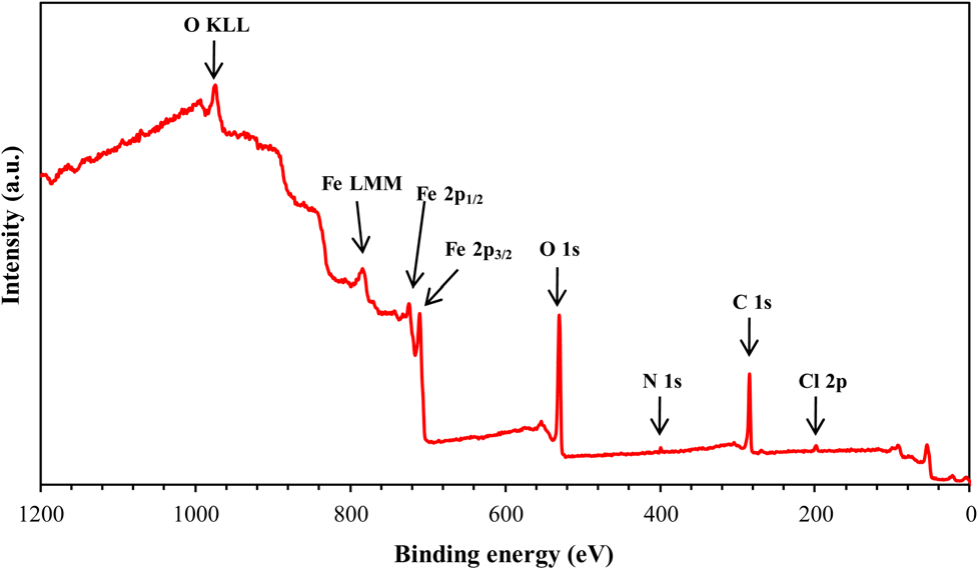
XPS spectrum of MR_2_/treated CS surface.

XPS analysis was employed to investigate the surface composition and chemical states of the materials at the interface. This technique provided insights into the adsorption behavior of the MR_1_ and MR_2_ inhibitors and the formation of a protective layer on metal surfaces. The XPS spectra of the surfaces treated with MR_1_ and MR_2_, presented in Fig. 9 and 10, reveal the presence of Fe, Cl, O, N and C atoms. These findings confirm the effective adsorption of the inhibitors and establishment of a protective layer on the metal surface.

**Fig. 11:**
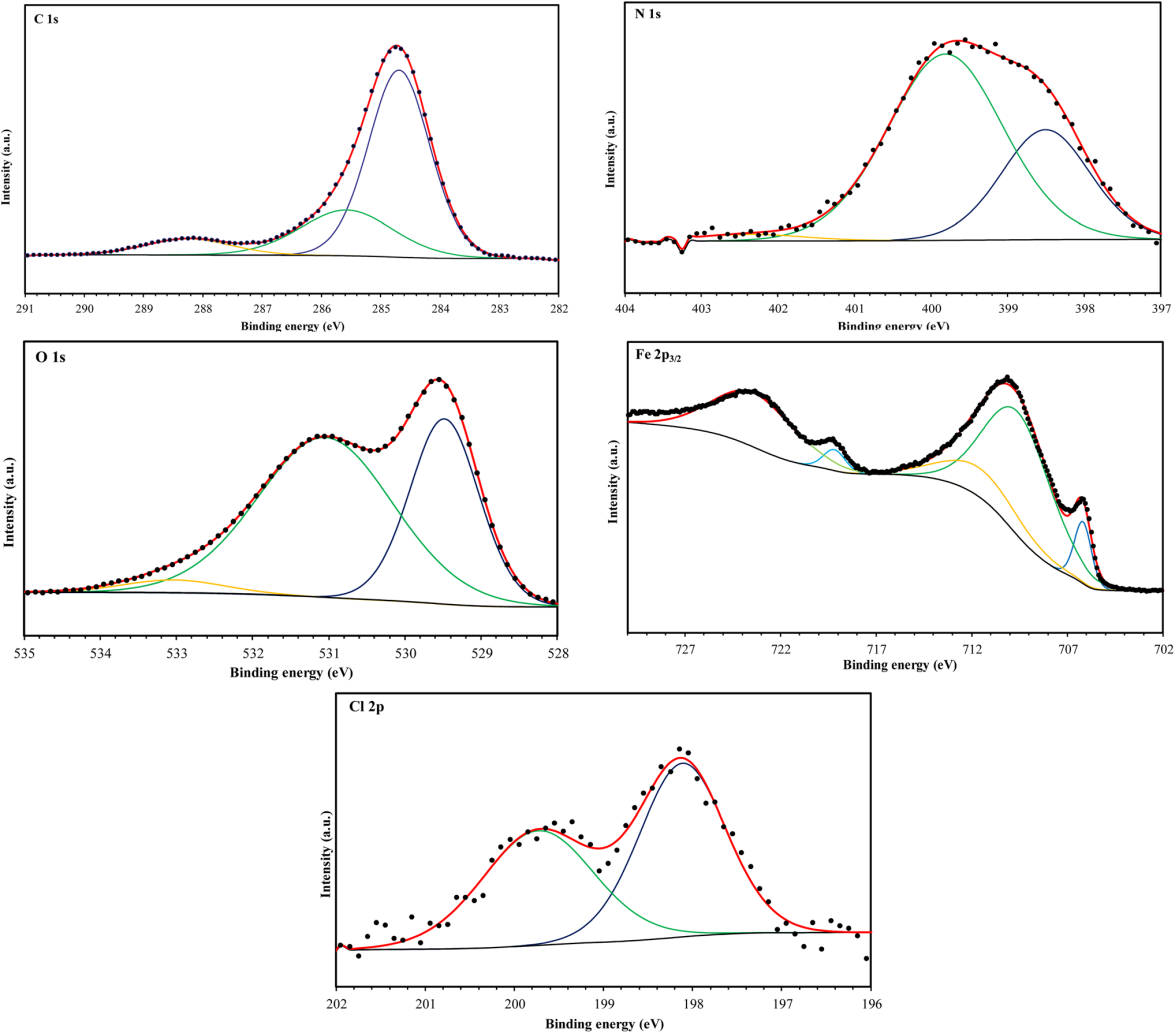
C 1s, N 1s, O 1s, Fe 2p, and Cl 2p XPS signals for MR_1_.

**Fig. 12:**
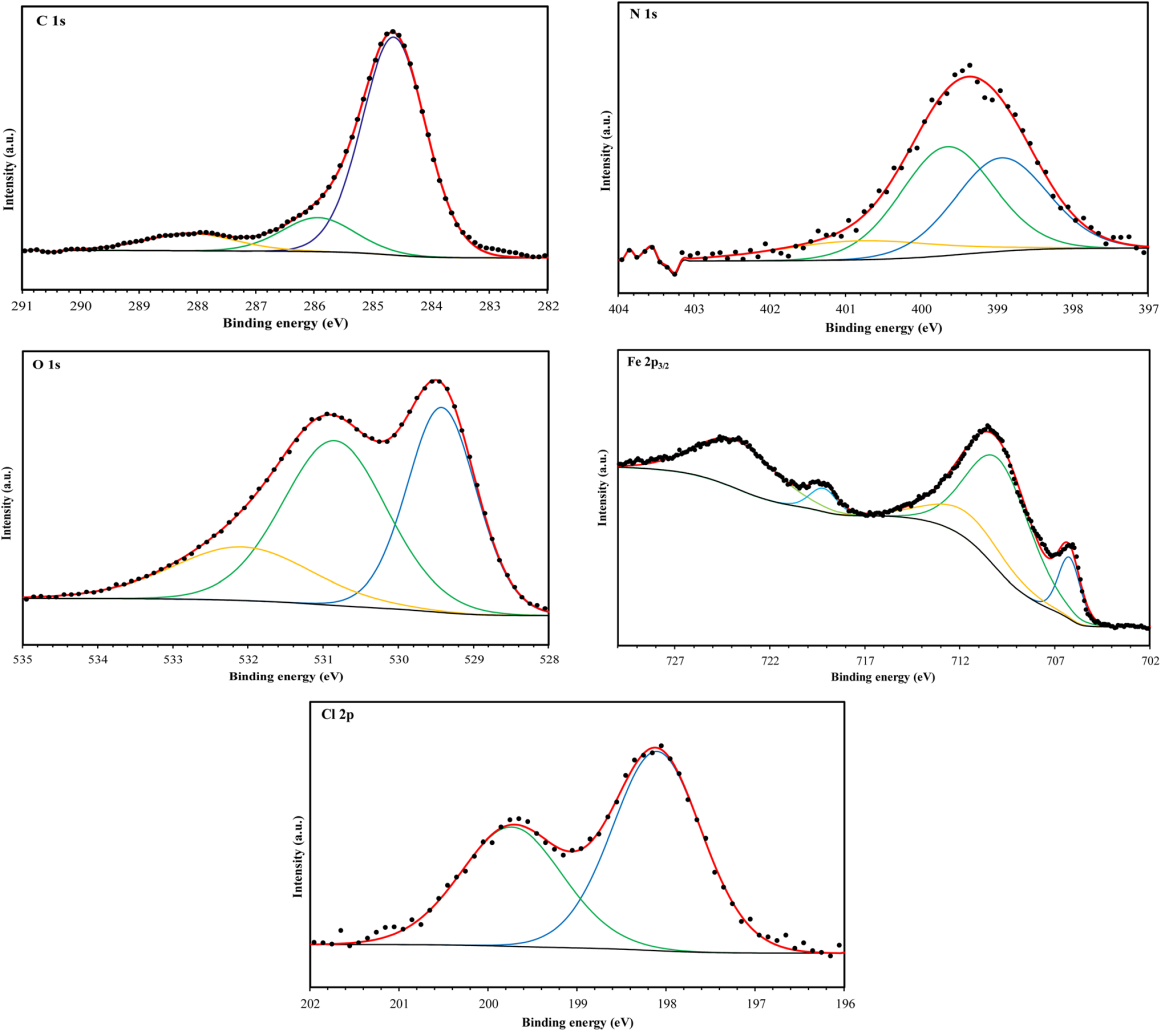
C 1s, N 1s, O 1s, Fe 2p, and Cl 2p XPS signals for MR_2_.

The high-resolution C 1s spectra (Fig. 9–12) exhibits three main peaks, corresponding to different C positions within the MR_1_ and MR_2_ structures. The first peak appears at 284.7 eV for MR_1_ and 284.6 eV for MR_2_. This dominant peak, accounting for 67% (MR_1_) and 77% (MR_2_) of the C 1s signal, is attributed to carbon atoms in C–C, C=C, and C–H bonds, which are characteristic of the aromatic and aliphatic hydrocarbons in the inhibitor structures. Secondly, the next peaks were appeared at 285.6 eV for MR_1_ and 285.9 eV for MR_2_, contributes 25% (MR_1_) and 14% (MR_2_) of the C 1s signal. These peaks are associated with C atoms bonded to electronegative atoms such as N and O, indicating the presence of C–N, C=N, and C–O bonds within the inhibitor structures. These functional groups play a crucial role in facilitating interaction between the metallic surface and Fe. The third peak is appeared at 288.2 eV for MR_1_ and 288.1 eV for MR_2_, corresponding to 8% (MR_1_) and 9% (MR_2_) of the C 1s signal, respectively. This peak is attributed to carbon atoms involved in C=O bonds and possibly C–N^+^ bonds. The presence of C–N^+^ suggests protonation of N atoms in the acidic environment, enhancing the adsorption of the inhibitors through electrostatic interactions.

The second XPS spectra for N 1s spectra (Fig. 9–12) reveal three N 1s peaks, showing their various positions within the inhibitor structures. The first peak, signaled at 398.5 eV for MR_1_ and 398.9 eV for MR_2_ is attributed to non-protonated N atoms in C–N and =N– bonds. This indicates that some N atoms remain non-protonated and are available for coordination with the metal surface. The second set of peaks, appearing at 399.8 eV for MR_1_ and 399.7 eV for MR_2_, confirms the presence of N–Fe bonds, signifying a direct interaction between N atoms with Fe. These signals suggest the chemisorption of the MR_1_ and MR_2_*via* N atoms, thereby contributing to the formation of a protective layer on the metal. Finally, the presence of protonated N atoms (=N^+^H–) is evidenced by the signals at the 401.4 eV for MR_1_, and 401.0 eV for MR_2_, enhancing their positive charge and interaction with negatively charged Cl^−^ ions on the surface.

The peak of O 1s spectra at 529.5 eV for MR_1_ and 529.4 eV for MR_2_ is associated with O^2−^ ions in iron oxides such as Fe_2_O_3_ and Fe_3_O_4_, indicating the formation of oxide layers during the corrosion process. Next O 1s peak observed at 531.1 eV for MR_1_ and 530.8 eV for MR_2_ corresponds to OH^−^ ions present in hydrous iron oxides like FeOOH and oxygen atoms involved in C=O, O–H, and C–O bonds within the inhibitors. This peak thus reflects both corrosion products and the adsorbed MR_1_ and MR_2_ onto the metal surface. Lastly, the O of adsorbed water molecules on the steel surface is detected at 533.0 eV for MR_1_ and 532.1 eV for MR_2_, confirming the presence of H_2_O on the surface.

The oxidation states of Fe at the surface were also analyzed. The Fe 2p spectrum reveals a peak at 706.2 eV, corresponding to metallic iron (Fe^0^), indicating regions that remain unoxidized. The second peak at 710.1 eV confirms the presence of Fe^3+^ species in iron oxides (Fe_2_O_3_) and oxyhydroxides (FeOOH), which are typical corrosion products forming a passive layer on the underlying metal. Finally, peaks at 713.9 eV for MR_1_ and 714.1 eV for MR_2_ are also characteristic of Fe^3+^ and may suggest the presence of FeCl_3_, indicating a potential interaction between iron and chloride ions from the HCl solution.

The chlorine (Cl 2p) spectra exhibit a peak at 198.1 eV (Cl 2p_3/2_) indicating that the Cl^−^ ions have interacted with iron to form FeCl_3_. The relatively low intensity of Cl peaks (0.34% for MR_1_ and 1.12% for MR_2_, as shown in Table 4) suggests that the inhibitors effectively reduce chloride adsorption, thereby mitigating its corrosive effect.

**Table 4:** XPS results for selected compounds

Element	Position (eV)	Assignment
MR_1_		
C 1s	288.2 (8%)	C–N^+^/C=O
	285.6 (25%)	C–O/C=N/C–N
	284.7 (67%)	C=C/C–C/C–H
N 1s	402.4 (2%)	–N^+^H
	399.8 (67%)	N–Fe
	398.5 (31%)	–N=/N–C structure
O 1s	529.5 (36%)	O^2−^ in Fe_2_O_3_
	531.1 (60%)	OH^−^ in FeOOH/OH/O–C/O=C
	533.0 (4%)	Adsorbed H_2_O
Cl 2p	199.7 (46%)	Cl 2p_1/2_
	198.1 (54%)	Cl 2p_3/2_
Fe 2p_3/2_	706.2 (9%)	Fe^0^
	710.1 (88%)	Fe^3+^ in Fe_2_O_3_ and in FeOOH
	713.9 (3%)	Satellite of Fe^3+^/FeCl_3_

MR_2_		
C 1s	284.6 (77%)	C=C/C–C/C–H/
	285.9 (14%)	C–N/C=N/C–O
	288.1 (9%)	C=O/C–N^+^
N 1s	401.0 (14%)	–N^+^H
	399.7 (46%)	N–Fe
	398.9 (40%)	N–C/–N= structure
O 1s	529.4 (37%)	O^2−^ in Fe_2_O_3_
	530.8 (44%)	OH^−^ in FeOOH/OH/O–C/O=C
	532.1 (19%)	Adsorbed H_2_O
Cl 2p	199.7 (41%)	Cl 2p_1/2_
	198.1 (59%)	Cl 2p_3/2_
Fe 2p_3/2_	706.2 (12%)	Fe^0^
	710.1 (86%)	Fe^3+^ in Fe_2_O_3_ and in FeOOH
	714.1 (2%)	Satellite of Fe^3+^/FeCl_3_

Table 5 shows a higher C content on MR_2_-treated steel (56.07%) compared to MR_1_ (49.51%) suggesting a more extensive inhibitor coverage and, consequently, enhanced protective performance. The low content of O implies less formation of iron oxides, possibly due to a more effective inhibition of the oxidation process by MR_2_. Lastly, the presence of N confirms the Fe–N interactions.^55–57^

**Table 5:** Percentage of elements on metal surface

Element	MR_1_ treated-steel	MR_2_ treated-steel
C	49.51	56.07
O	35.46	31.77
N	1.54	1.38
Cl	0.34	1.12
Fe	13.14	9.66

### 3.8. Theoretical studies

#### 3.8.1. DFT analysis

**Fig. 13:**
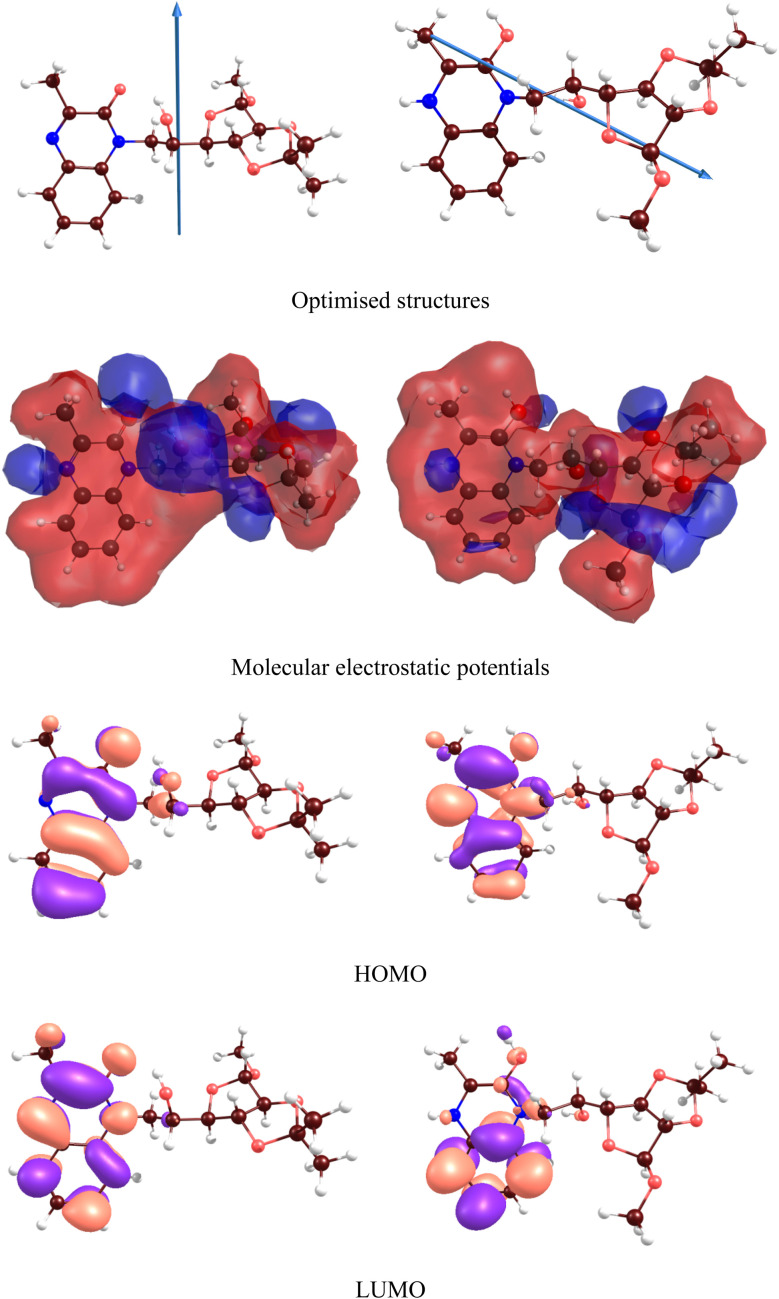
DFT results for (MR_1_, left) and protonated (MR_1_-H, right) forms.

**Fig. 14:**
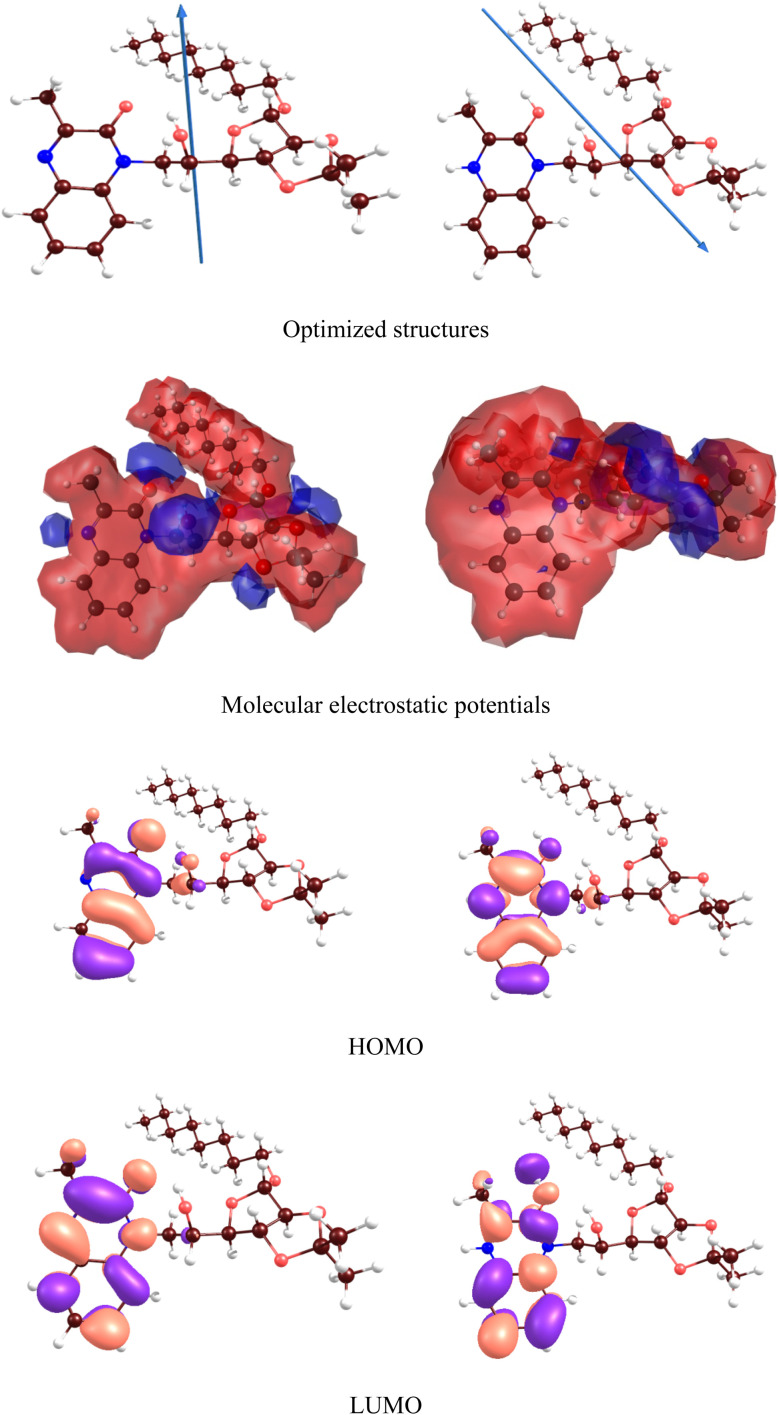
DFT results for (MR_2_, left) and protonated (MR_2_-H, right) forms.

Fig. 13 and 14 respectively illustrate the optimized structures, molecular electrostatic potentials and frontier molecular orbitals of the MR_1_ and MR_2_ inhibitors in aqueous phase. The HOMO and LUMO energies can be listed in Table 6. Both frontier orbitals are located near two hexagonal rings of the inhibitor, and their. Is significantly influenced by protonation. Table 6 also includes the estimated values of the ionization potential (IP) and electron affinity (EA). Instead of using the widely accepted Koopmans' theorem or empirical formulas^58^ typically employed to estimate IP and EA values from HOMO and LUMO energies, we calculated these values as (IP = *E*(M^+^) − *E*(M^0^); EA = *E*(M^0^) − *E*(M^−^)). Here *E*(M^0^), *E*(M^+^) and *E*(M^−^) represent the total energies of the neutral, positively charged, and negatively charged molecules, respectively.

**Table 6:** DFT results for (MR_1_ and MR_2_) and protonated (MR_1_-H and MR_2_-H) molecules: *E*_HOMO_ and *E*_LUMO_ (eV), HOMO–LUMO gaps (eV), dipole moments DM (Debye), ionization potentials IP and electron affinity EA (eV), electronegativity *χ* (eV), global hardness *η* (eV) and softness *S* (eV^−1^), electron transfer Δ*N* and back-donation energies *E*_bd_ (eV)

Parameter	MR_1_	MR_1_-H	MR_2_	MR_2_-H
*E*_HOMO_	−6.36	−4.47	−6.34	−4.55
*E*_LUMO_	−1.98	−0.09	−1.95	−0.17
Gap	4.38	4.38	4.39	4.38
DM	3.84	5.41	3.57	5.47
IP	6.93	4.50	7.14	4.54
EA	2.05	−0.45	2.06	−0.67
*χ*	4.49	2.03	4.60	1.93
*η*	2.19	2.19	2.20	2.19
*S*	0.46	0.46	0.46	0.46
Δ*N*	0.08	0.64	0.05	0.66
*E*_bd_	−0.55	−0.55	−0.55	−0.55

Based on IP and EA, it was derived other relevant chemistry descriptors of inhibitors reactivity, following the methodology outlined in.^59^ Electronegativity (*χ*), global hardness (*η*) and softness (*S*), electron transfer Δ*N* and back-donation energy *E*_bd_ were also obtained and are presented in Table 6. The energy levels of the frontier molecular orbitals (*E*_HOMO_ and *E*_LUMO_) reveal that protonation raises the HOMO and LUMO levels, denoting greater chemical reactivity for MR_1_-H and MR_2_-H but unchanged HOMO–LUMO energy gaps (∼4.38–4.39 eV) for each protonated form showing similar chemical stability. The dipole moment, which is related to the molecular polarity of the selected compounds, increases from protonation (MR_1_: 3.84 to 5.41 D; MR_2_: 3.57 to 5.47 D) and indicates that the protonated forms can interact further with the polar surface of steel, thus creating stronger adsorption. Protonation significantly lowers both the IP and the EA, with the EA exhibiting negative values, which illustrates a greater tendency for these protonated molecules to donate electrons to the Fe surface.

Moreover, MR_1_-H and MR_2_-H show superior charge transfer interactions with the Fe surface, which is essential when considering the coating's effectiveness in corrosion inhibition. The electronegativity of neutral forms of both molecules nearly equals that of the steel surface (4.82 eV (ref. 59)) which can suggest little to no transfer of electrons.

Comparing the calculated electronegativity of the protonated forms demonstrates an enlivened Δ*N* due to the lowers approximating ∼1.9–2.0 eV MR_1_: 0.08 to 0.64; MR_2_: 0.05 to 0.66.

These results that the protonated forms of MR_1_-H and MR_2_-H exhibit a stronger tendency to donate electrons to the Fe surface, thereby forming stronger coordination complexes on this surface. The nearly identical HOMO–LUMO gaps of all the molecules lead to similar values of *η* and *E*_bd_, as shown in Table 6. The values of *η* and *S* values remain consistent across all molecules, suggesting comparable reactivity. Furthermore, the *E*_bd_ remains stable at −0.55 eV, indicating stable interactions involving electron back-donation from the metal to the inhibitors' antibonding orbitals, further stabilizing the inhibitor-metal complex.

**Fig. 15:**
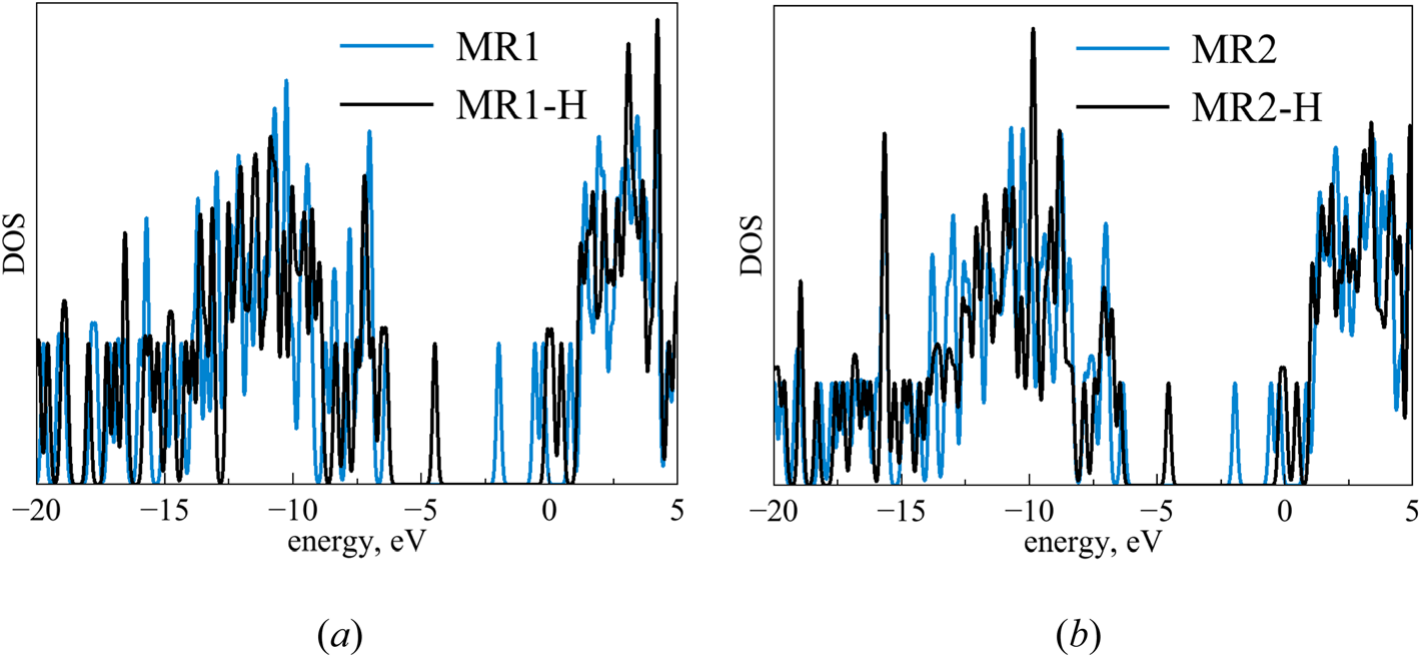
DOS for MR_1_ (a) and MR_2_ (b) inhibitors in both neutral and protonated forms (FWHM = 0.2 eV).

To reveal the impact of protonation on the electronic structures of the inhibitors, we have depicted the electronic density of states (DOS) for both the neutral and protonated forms of the inhibitors, as shown in Fig. 15. It is evident that protonation induces significant changes in the DOS, particularly near the gap and gap shifting. To assess local reactivity, Fukui indices were calculated for all inhibitor atoms. The Fukui indices for nucleophilic (*f*^+^) and electrophilic (*f*^−^) attacks were defined as (*f*^+^ = *D*(M^−^) − *D*(M^0^); *f*^−^ = *D*(M^0^) − *D*(M^+^)).

**Fig. 16:**
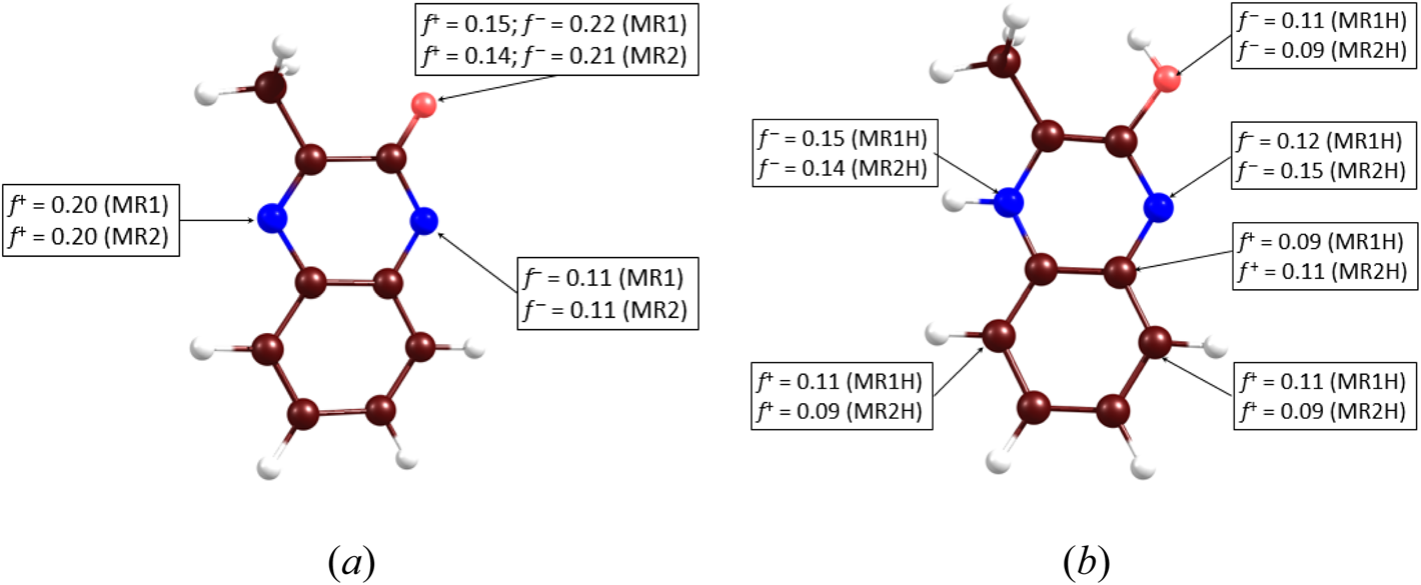
Fukui results for MR_1_ and MR_2_ molecules in neutral (a) and protonated forms (b) (*f*^+^ and *f*^−^ are higher than 0.1 e bohr^−3^).

The electron density at the considered nucleus of the neutral, positively charged, and negatively charged inhibitor molecule is denoted as *D*(M^0^), *D*(M^+^) and *D*(M^−^). Atoms with *f*^+^ and *f*^−^ values greater than 0.1 e bohr^−3^ are highlighted in Fig. 16.

#### 3.8.2. MD analysis

**Fig. 17:**
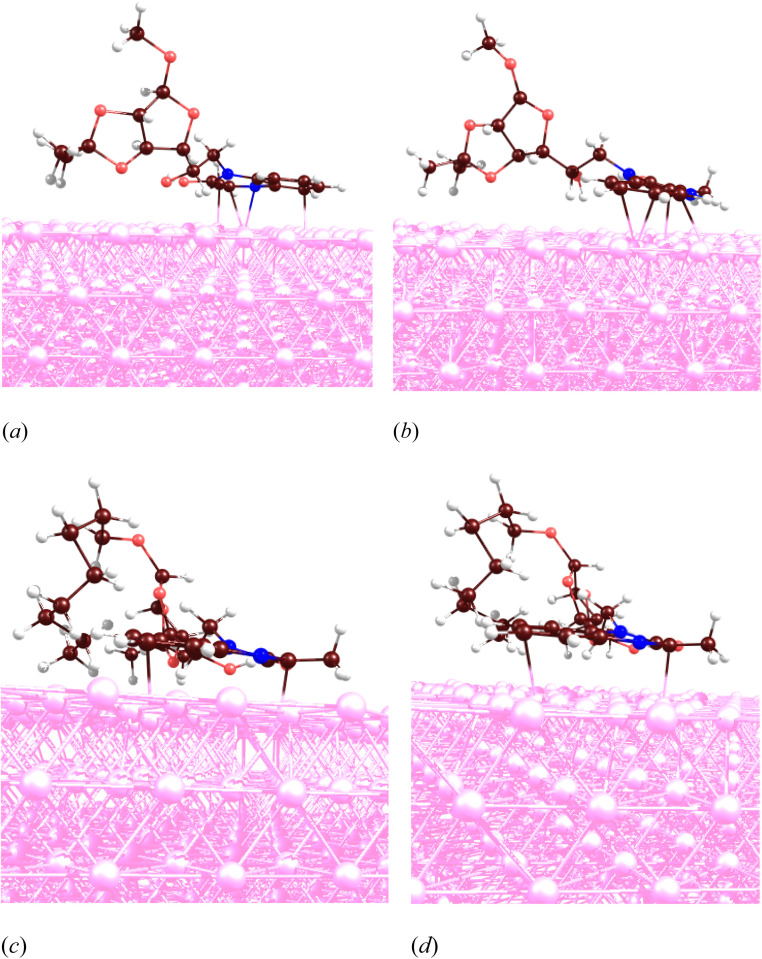
Metal-inhibitor interactions: MR_1_ (a), MR_1_-H (b), MR_2_ (c) and MR_2_-H (d).

**Fig. 18:**
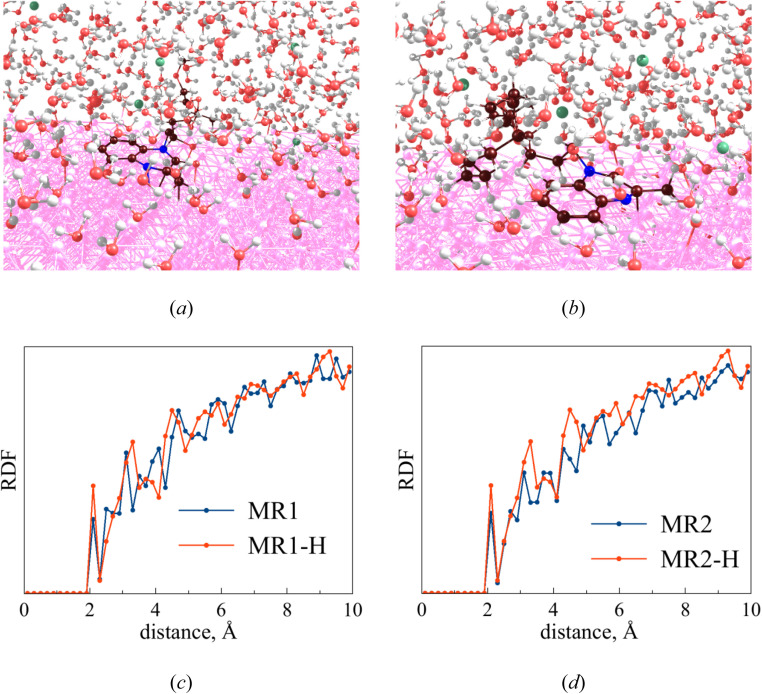
Metal-inhibitor interactions: MR_1_ (a) and MR_2_ (b) in a corrosive media. RDF plots: MR_1_ (c) and MR_2_ (d).

The MD analyses demonstrated that the inhibitors MR_1_ and MR_2_ adsorbs onto the Fe(110) surface at the same position, as illustrated in Fig. 17. It is evident that two hexagonal rings, including heteroring of nitrogen atoms, along with frontier molecular orbitals, attract the molecules to the steel surface. The MD approach was employed to observe the inhibitor's temporal evolution on the steel surface within a corrosive media. Following a 1 ns simulation, the molecules were successfully adsorbed on the steel surface, as shown in Fig. 18. The radial distribution function (RDF) for the Fe and inhibitor atoms was computed to describe the distance distribution between the iron and the inhibitor, as shown in Fig. 18. The initial RDF peak near 2 Å confirms that the inhibitor remained adsorbed to the steel surface throughout the simulation. No significant differences in the positions and RDF functions of the neutral inhibitor and protonated forms were identified, yet the increased polarity of protonated forms led them to having greater electrostatic interactions and hence higher electrons-donation ability.^60–62^ Molecular dynamics (MD) simulations have provided more insight into the adsorption behavior of MR_1_ and MR_2_ inhibitors on the Fe(110) steel surface, mostly in the molecular orientation, the preferred adsorption positions, and their active adsorption positions. The MR_1_ and MR_2_ molecules, as well as their protonated forms (MR_1_-H and MR_2_-H), all adsorb flat and parallel to the Fe surface.

At parallel positions, this parallel increases surface contact giving maximum adsorption strength and overall coverage of the surface which is expected to be a good corrosion inhibition. The principal adsorption sites of these molecules are centered on heteroatoms such as nitrogen (–N= and –NH–) and oxygen (C=O, –OH) as well as the conjugated π-systems of the quinoxaline aromatic rings. Representatives of these functional groups are a rich source of electron, making them suitable for establishing coordination with the iron surface. The presence of lone pair electrons on N and O atoms also favors coordination bond formation or donor–acceptor interactions with the vacant d-orbitals of Fe atoms. In addition, the alkyl chains of MR_1_ and MR_2_ form a hydrophobic protective film that adds additional shielding to the metal surface from the corrosive environment. The two hexagonal rings contained in the quinoxaline core portion of MR_1_ and MR_2_ perform vital functions to immobilize each molecule metal surface. They are in the region of frontier molecular orbitals (HOMO and LUMO) that are largely sitting on aromatic systems and surrounding nitrogen atoms. The overlapping spatial overlap interactions facilitate closer electronic coupling at adsorption interface.

### 3.9. Comparative study

Table 7 presents a comparison between the inhibitors studied here and other inhibitors reported in literature.

**Table 7:** Performance of inhibitors MR_1_ and MR_2_ with related compounds described in literature

Structure of inhibitors	HCl concentration	Inhibitor concentration	IE (%)	Reference
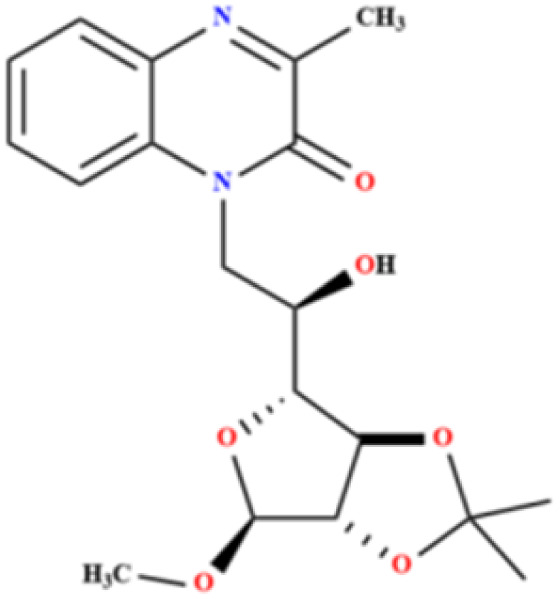	1.0 M	10^−3^ M	93.2	This work
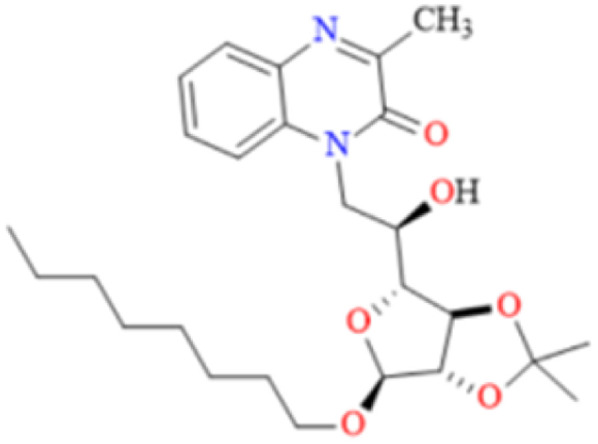	1.0 M	10^−3^ M	93.7	This work
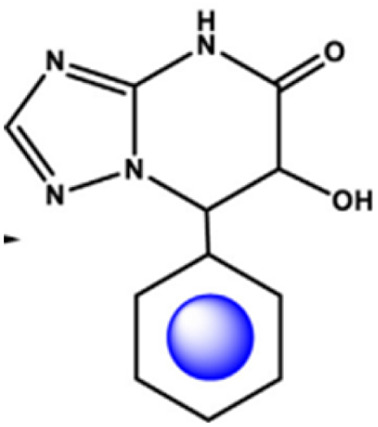	1.0 M	10^−3^ M	96.20	49
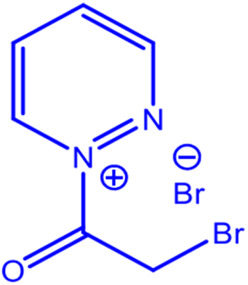	1.0 M	10^−3^ M	81.45	63
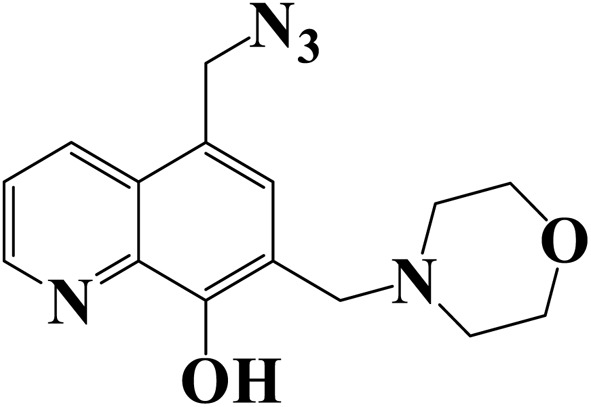	1.0 M	10^−3^ M	90	64

Table 7 that our inhibitors display intensive corrosion inhibition efficiency for triazolopyridine and ionic liquid in the same concentration level and at ambient temperature. Nevertheless, our compounds found an efficiency almost that is almost similar to 8-hydroxyquinoline. The potential functionalities computed for the organic compounds MR_1_ and MR_2_ would allow them, as it has molecular structure,^65^ with two quinoxalines a attached together through d-mannose derived. As sited above, this configuration results in many hydroxyl groups per molecule and capable of forming a stronger adsorption onto steel surface.

## 4. Conclusion

The key findings of this study show that two quinoxaline derivatives, MR_1_ and MR_2_, were synthesized from d-mannose, and their structures were confirmed by ^13^C-NMR, and ^1^H-NMR spectroscopy. At 0.4 g per L concentration of these inhibitors exhibited significant corrosion inhibition efficiencies of 95.3% for MR_1_ and 94.8% for MR_2_. Both inhibitors demonstrated a mixed inhibition mechanism, leading to a substantial reduction in corrosion current density (*i*_corr_), with a minimum value of 51.4 μA cm^−2^ for MR_2_. Electrochemical impedance spectroscopy (EIS) analysis revealed an increase in polarization resistance (*R*_p_), reaching a maximum of 343.5 Ω cm^−2^ for MR_2_, indicating improved surface protection. The slight shift in corrosion potential (*E*_corr_) (<22 mV) suggests that the inhibition mechanism is primarily mixed-type, with minimal influence on anodic and cathodic processes. Additionally, the decrease in double-layer capacitance (*C*_dl_) with increasing inhibitor concentration suggests a moderate displacement of water molecules by the inhibitor at the metal surface.

The adsorption of MR_1_ and MR_2_ inhibitors on the steel surface follows the Langmuir adsorption isotherm model, with an excellent fit (*R*^2^ = 0.9999). The calculated Δ*G*_ads_ values of −37.7 kJ mol^−1^ for MR_2_ and -37 kJ mol^−1^ for MR_1_ indicate spontaneous adsorption. These results, along with XPS data, highlight strong interactions between the inhibitors and the steel surface, suggesting that the adsorption mechanism involves both physisorption and chemisorption. The formation of a protective inhibitor layer led to a noticeable smoothing of the steel surface treated with MR_2_, as observed by scanning electron microscopy (SEM). The contact angles for MR_1_ and MR_2_ increased to 121.2° and 129.5°, respectively, indicating enhanced surface hydrophobicity, which is further reinforced by the alkyl chains present in the inhibitors. XPS analysis confirmed the formation of protective layers, with significant shifts in Fe, N, and O peaks, indicating effective adsorption.

XPS data also revealed the presence of Fe, C, N, and O on the treated surfaces, with a reduction in oxygen concentration to 31.77% for MR_2_, providing optimal protection. The HOMO–LUMO gap of approximately 4.38 eV for both MR_1_ and MR_2_ attests to their chemical stability and adequate reactivity. Although their electrical characteristics are similar, the longer alkyl chain of MR_2_ promotes enhanced physical adsorption. Molecular dynamics simulations confirmed stable adsorption of MR_1_ and MR_2_ on the Fe(110) surface, with RDF peaks indicating a proximity of 2 Å. A significant reduction in the corrosion rate of steel in 1.0 M HCl solution validated the effectiveness of both inhibitors. Corrosion inhibition by d-mannose derivatives presents an eco-friendly and environmentally benign approach. Furthermore, the protonated forms of MR_1_ and MR_2_ exhibit high electron transfer capacity (Δ*N* close to 0.66), further strengthening their interaction with the metal surface.

## Data availability

All cited data can be found in the manuscript and the ESI files.†

## Conflicts of interest

The authors state that the publication of this paper is not influenced by any conflicts of interest.

## Supplementary Material

RA-015-D5RA00835B-s001
