# Supplementary material for: New sugar-derived compounds as inhibitors of carbon steel against corrosion in acid solutions: experimental analyses and theoretical approaches
Source: RSC Adv. 2025 May 19;15(21):16570–87. doi: 10.1039/d5ra00835b (PMC12086530; doi:10.1039/d5ra00835b)
Supplement: RA-015-D5RA00835B-s001 [file RA-015-D5RA00835B-s001.pdf]

Supplementary Information (SI) for RSC Advances.
This journal is © The Royal Society of Chemistry 2025

New sugar-derived compounds as inhibitors of carbon steel against corrosion in acid solutions: Experimental analyses and theoretical approaches

M. Rbaa [1,2,3], A. Barrahi [3], R. Seghiri [4,5], Konstantin P. Katin[6], Elyor Berdimurodov [7,8,9], Hatem A. Abuelizz [10], C. Jama [11], F. Bentiss [11,12], B. Lakhrissi [2], A. Zarrouk [3]

(1) The Higher Institute of Nursing Professions and Health Techniques of Casablanca, P.O. Box 20250, Casablanca, Morocco.
(2) Laboratory of Organic Chemistry, Catalysis and Environment, Faculty of Sciences, Ibn Tofail University, PO Box 133, 14000, Kenitra, Morocco.
(3) Laboratory of Materials, Nanotechnology, and Environment, Faculty of Sciences, Mohammed V University in Rabat, P.O. Box 1014, Agdal-Rabat, Morocco.
(4) Ecole Nationale Supérieure de Chimie de Kenitra (ENSCK), Ibn Tofail University, Kenitra, Morocco.
(5) Laboratory of Advanced Materials and Process Engineering, Faculty of Sciences, Ibn Tofail University, Kenitra, Morocco.
(6) National Research Nuclear University "MEPhI", KashirskoeShosse 31, Moscow 115409, Russian Federation.
(7) Faculty of Chemistry, National University of Uzbekistan, Tashkent, 100034, Uzbekistan.
(8) Department of Pharmacy and Chemistry, Alfraganus University, Tashkent,100190, Uzbekistan.
(9) Chemistry and Physics,Western Caspian University, Baku, AZ-1001, Azerbaijan.
10) Department of Pharmaceutical Chemistry, College of Pharmacy, King Saud University, PO Box 2457, Riyadh, 11451, Saudi Arabia.
(11) Univ. Lille, CNRS, INRAE, Centrale Lille, UMR 8207, - UMET - Unité Matériaux et Transformations, F-59000 Lille, Lille, France.
(12) Laboratory of Catalysis and Corrosion of Materials, Faculty of Sciences, Chouaib Doukkali University,PO Box 20, M-24000 El Jadida, Morocco.

Corresponding authors
Prof. Dr. Abdelkader Zarrouk
Email:azarrouk@gmail.com (AZ)
Phone:00212665201397
Scopus Author ID: 36125763200
https://orcid.org/0009-0003-2175-7280
Prof. Dr. Mohamed Rbaa
Email: mohamed.rbaa10@gmail.com (MR)
Phone:00212702099423
Scopus Author ID:57096113600
https://orcid.org/0000-0003-1235-890X

Spectroscopic analysis of organic compounds MR_1 and MR_2

1-N-(6-deoxy-2,3-O-isopropylidene-α-D-mannofuranoside of methyl-6-yl)-3-methylquinoxalinones (MR_1)

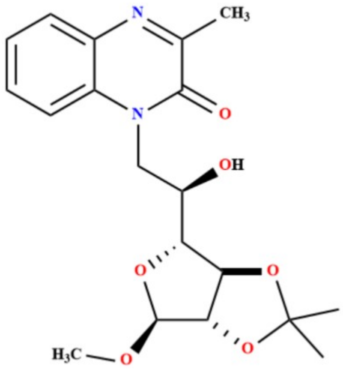

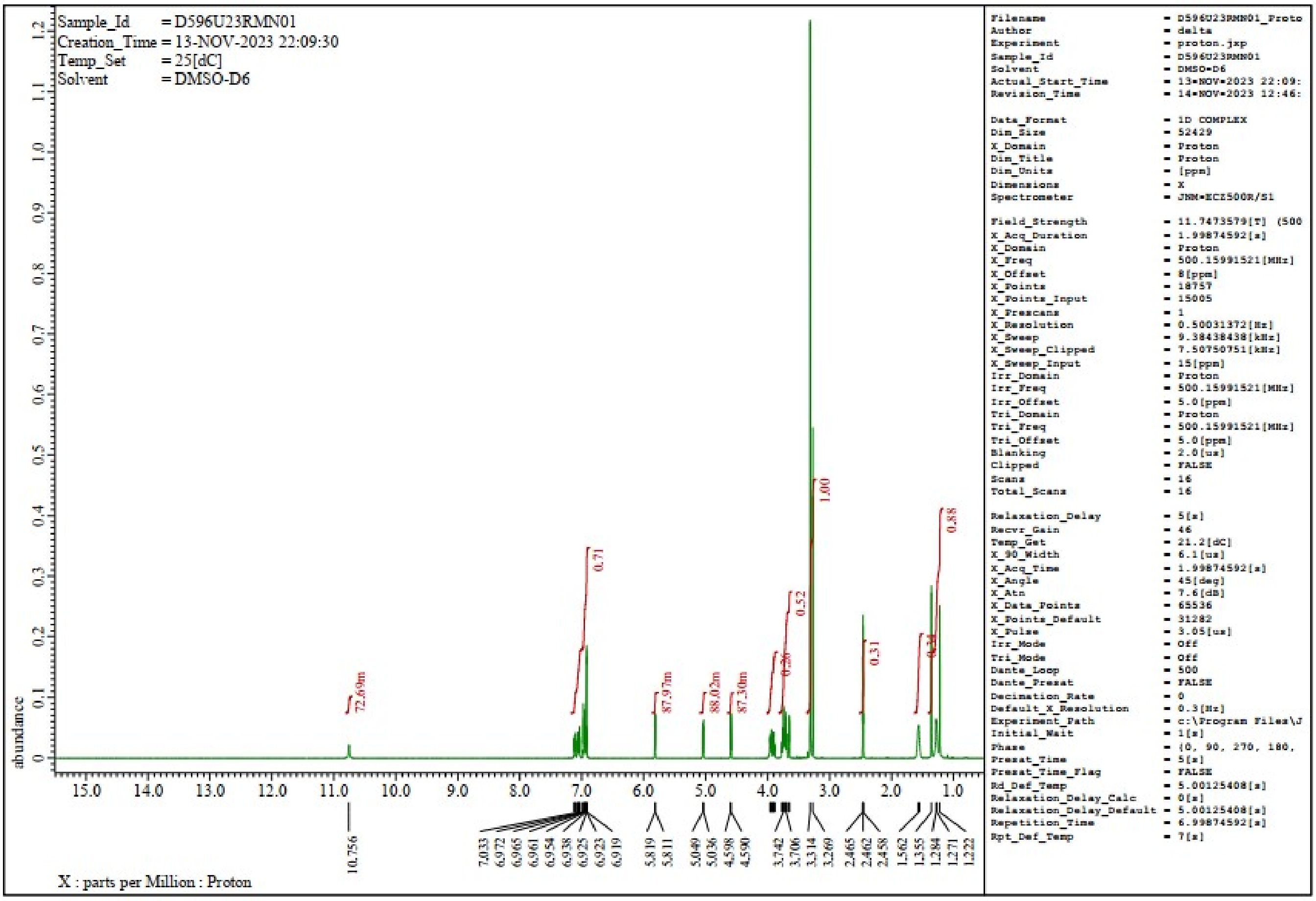

Sample_Id = D596U23RMN01
Creation_Time = 13-NOV-2023 22:09:30
Temp_Set = 25[dC]
Solvent = DMSO-D6
abundance
X : parts per Million : Proton

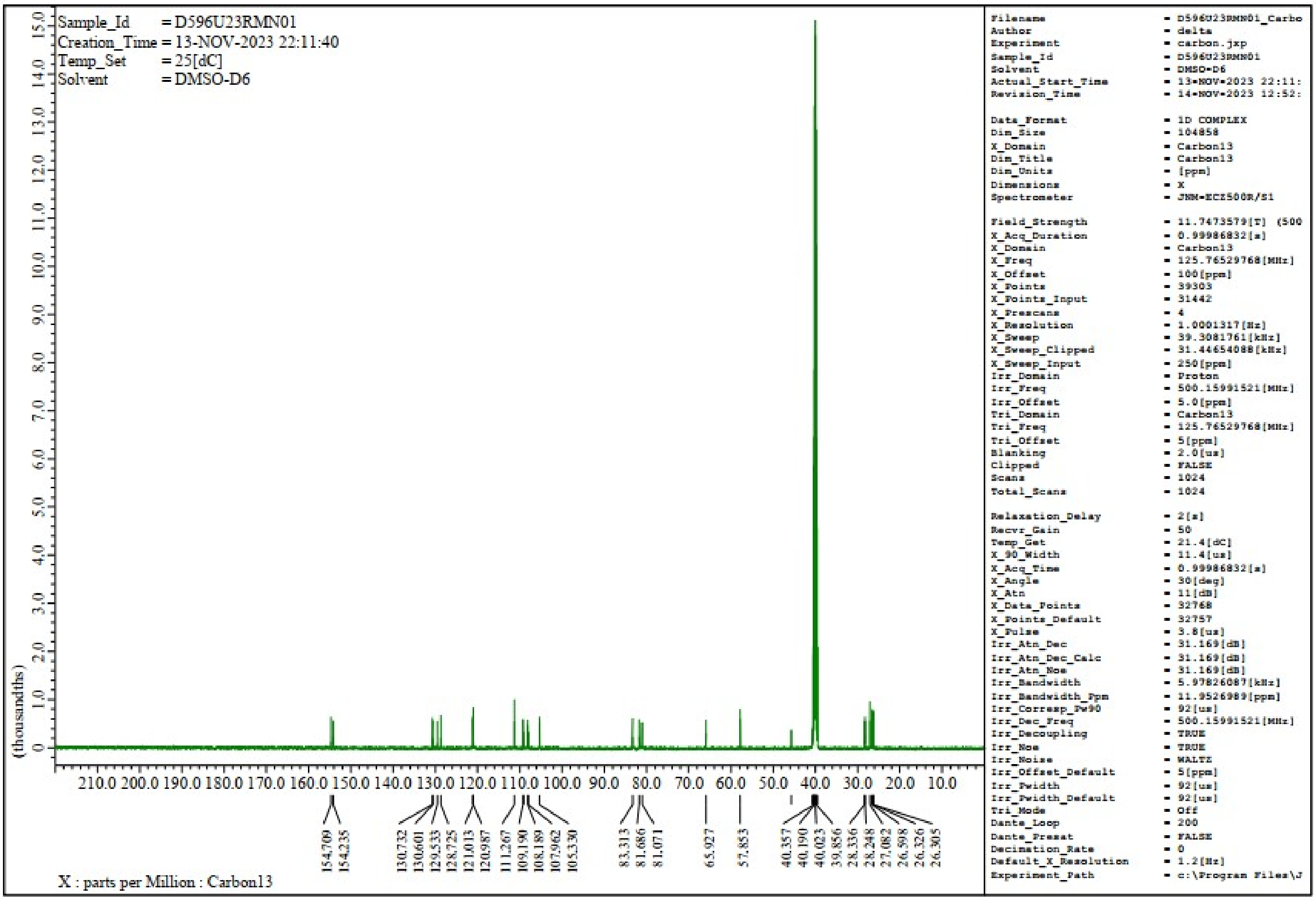

Sample_Id = D596U23RMN01
Creation_Time = 13-NOV-2023 22:11:40
Temp_Set = 25[dC]
Solvent = DMSO-D6
(thousandths)
X : parts per Million : Carbon13

1-N-(6-deoxy-2,3-O-isopropylidene-α-D-mannofuranoside of octyl-6-yl)-3-methylquinoxalinones (MR$_2$)

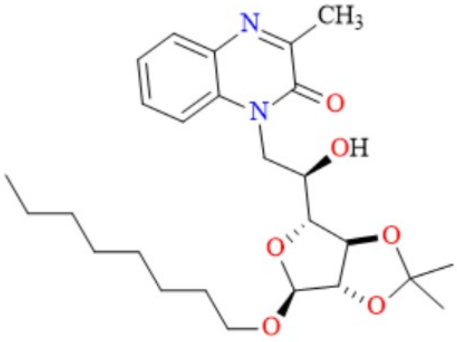

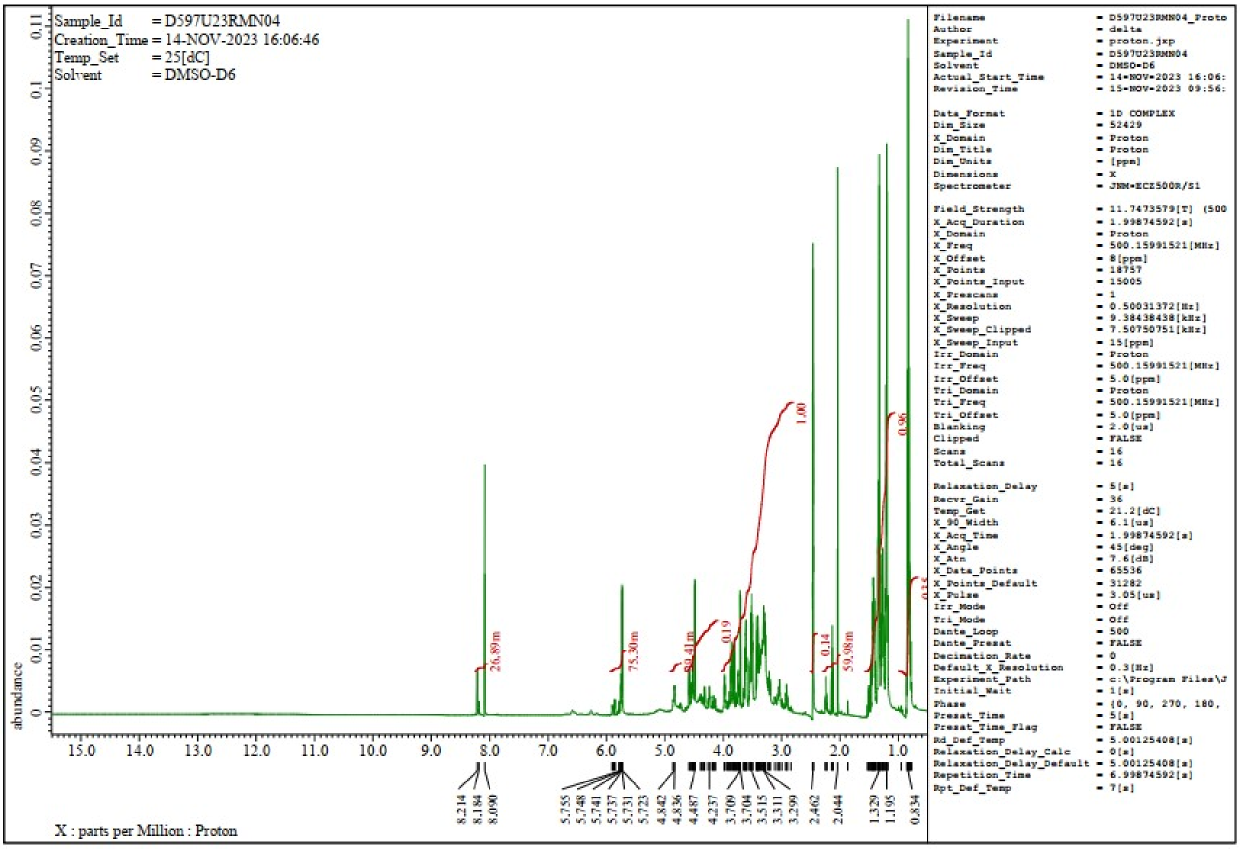

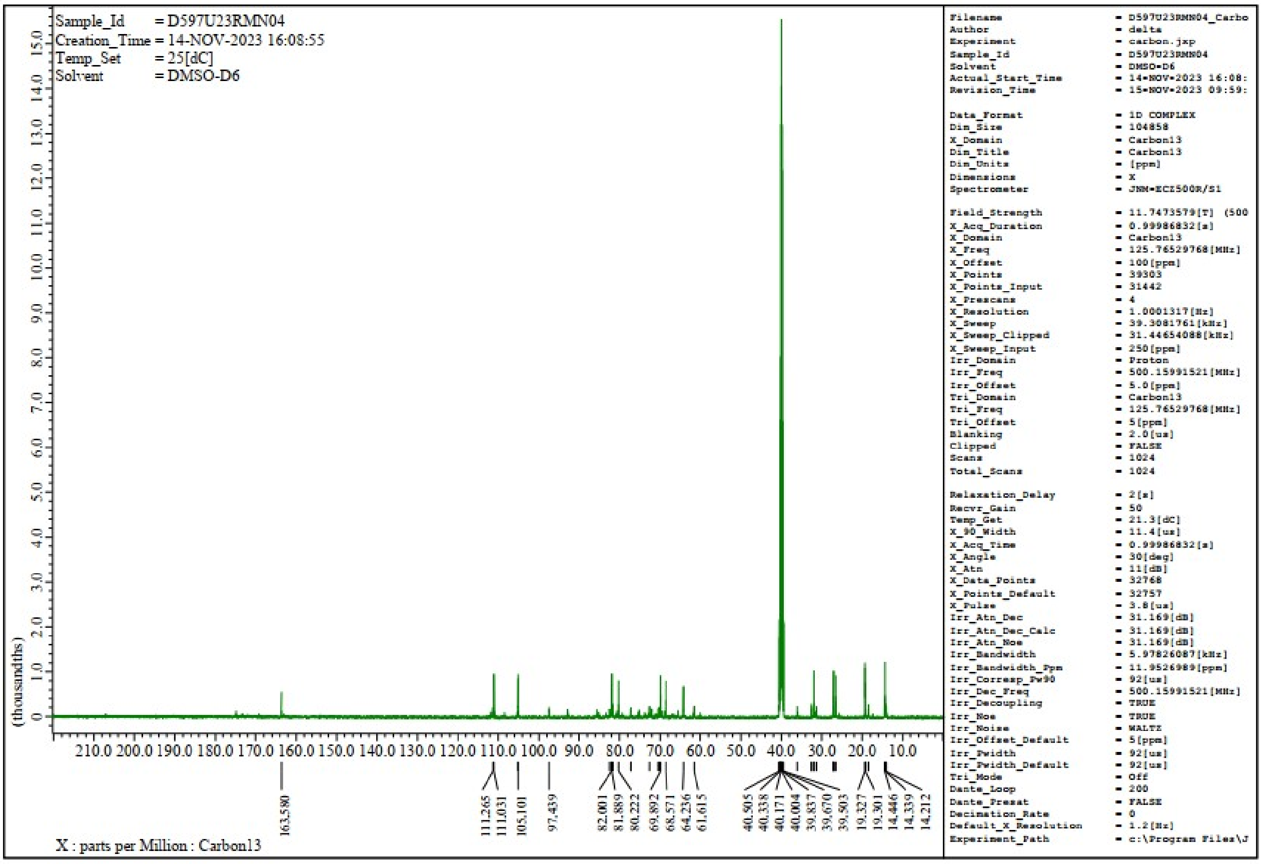
Sample_Id = D597U23RMN04
Creation_Time = 14-NOV-2023 16:08:55
Temp_Set = 25[dC]
Solvent = DMSO-D6
(thousandths)
X : parts per Million : Carbon13
163.580
111.265
111.031
105.101
97.439
82.001
81.889
80.222
69.892
68.571
64.236
61.615
40.505
40.338
40.171
40.004
39.837
39.670
39.503
19.327
19.301
14.446
14.339
14.212
Filename = D597U23RMN04_Carbo
Author = delta
Experiment = carbon.jxp
Sample_Id = D597U23RMN04
Solvent = DMSO-D6
Actual_Start_Time = 14-NOV-2023 16:08:
Revision_Time = 15-NOV-2023 09:59:
Data_Format = 1D COMPLEX
Dim_Size = 104858
X_Domain = Carbon13
Dim_Title = Carbon13
Dim_Units = [ppm]
Dimensions = X
Spectrometer = JNM-ECZ500R/S1
Field_Strength = 11.7473579[T] (500
X_Acq_Duration = 0.99986832[s]
X_Domain = Carbon13
X_Freq = 125.76529768[MHz]
X_Offset = 100[ppm]
X_Points = 39303
X_Points_Input = 31442
X_Prescans = 4
X_Resolution = 1.0001317[Hz]
X_Sweep = 39.3081761[kHz]
X_Sweep_Clipped = 31.44654088[kHz]
X_Sweep_Input = 250[ppm]
Irr_Domain = Proton
Irr_Freq = 500.15991521[MHz]
Irr_Offset = 5.0[ppm]
Tri_Domain = Carbon13
Tri_Freq = 125.76529768[MHz]
Tri_Offset = 5[ppm]
Blanking = 2.0[us]
Clipped = FALSE
Scans = 1024
Total_Scans = 1024
Relaxation_Delay = 2[s]
Recvr_Gain = 50
Temp_Get = 21.3[dC]
X_90_Width = 11.4[us]
X_Acq_Time = 0.99986832[s]
X_Angle = 30[deg]
X_Atn = 11[dB]
X_Data_Points = 32768
X_Points_Default = 32757
X_Pulse = 3.8[us]
Irr_Atn_Dec = 31.169[dB]
Irr_Atn_Dec_Calc = 31.169[dB]
Irr_Atn_Noe = 31.169[dB]
Irr_Bandwidth = 5.97826087[kHz]
Irr_Bandwidth_Ppm = 11.9526989[ppm]
Irr_Corresp_Pw90 = 92[us]
Irr_Dec_Freq = 500.15991521[MHz]
Irr_Decoupling = TRUE
Irr_Noe = TRUE
Irr_Noise = WALTZ
Irr_Offset_Default = 5[ppm]
Irr_Pwidth = 92[us]
Irr_Pwidth_Default = 92[us]
Tri_Mode = Off
Dante_Loop = 200
Dante_Presat = FALSE
Decimation_Rate = 0
Default_X_Resolution = 1.2[Hz]
Experiment_Path = c:\Program Files\J
